# *Lecanosticta acicola*: A growing threat to expanding global pine forests and plantations

**DOI:** 10.1111/mpp.12853

**Published:** 2019-07-15

**Authors:** Ariska van der Nest, Michael J. Wingfield, Josef Janoušek, Irene Barnes

**Affiliations:** ^1^ Forestry and Agricultural Biotechnology Institute (FABI), Department of Biochemistry, Genetics and Microbiology University of Pretoria Pretoria 0002 South Africa; ^2^ Phytophthora Research Center Mendel University in Brno Brno Czech Republic

**Keywords:** brown spot needle blight, *Lecanosticta acicola*, *Lecanosticta* species, *Mycosphaerella dearnessii*, pine pathogen, *Pinus* spp

## Abstract

*Lecanosticta acicola* causes brown spot needle blight (BSNB) of *Pinus* species. The pathogen occurs mostly in the Northern Hemisphere but has also been reported in Central America and Colombia. BSNB can lead to stunted growth and tree mortality, and has resulted in severe damage to pine plantations in the past. There have been increasingly frequent new reports of this pathogen in Europe and in North America during the course of the past 10 years. This is despite the fact that quarantine practices and eradication protocols are in place to prevent its spread.

**Taxonomy:**

Kingdom Fungi; Phylum Ascomycota; Subphylum Pezizomycotina; Class Dothideomycetes; Subclass Dothideomycetidae; Order Capniodales; Family Mycosphaerellaceae; Genus *Lecanosticta*.

**Host range and distribution:**

*Lecanosticta* spp. occur on various *Pinus* species and are found in North America, Central America, South America (Colombia), Europe as well as Asia.

**Disease symptoms:**

Small yellow irregular spots appear on the infected pine needles that become brown over time. They can be surrounded by a yellow halo. These characteristic brown spots develop to form narrow brown bands that result in needle death from the tips down to the point of infection. Needles are prematurely shed, leaving bare branches with tufts of new needles at the branch tips. Infection is usually most severe in the lower parts of the trees and progresses upwards into the canopies.

**Useful websites:**

The EPPO global database providing information on *L. acicola* (https://gd.eppo.int/taxon/SCIRAC)

Reference genome of *L. acicola* available on GenBank (https://www.ncbi.nlm.nih.gov/genome/?term=Lecanosticta+acicola)

JGI Gold Genome database information sheet of *L. acicola* sequenced genome (https://gold.jgi.doe.gov/organism?xml:id=Go0047147)

## Introduction

*Lecanosticta acicola* is an ascomycete fungus that causes a disease of *Pinus* spp. known as brown spot needle blight (BSNB). The pathogen was first described by de Thümen (1878) and it owes its notoriety to a disease problem that arose in the southeastern USA on *Pinus palustris*, better known as long leaf pine in that area (Siggers, 1932). This tree species, which is highly susceptible to infection, is peculiar in having a so‐called ‘grass’ stage during the first five years of its growth. This mass of young needles provides a favourable environment for infection to occur.

**Figure 1:**
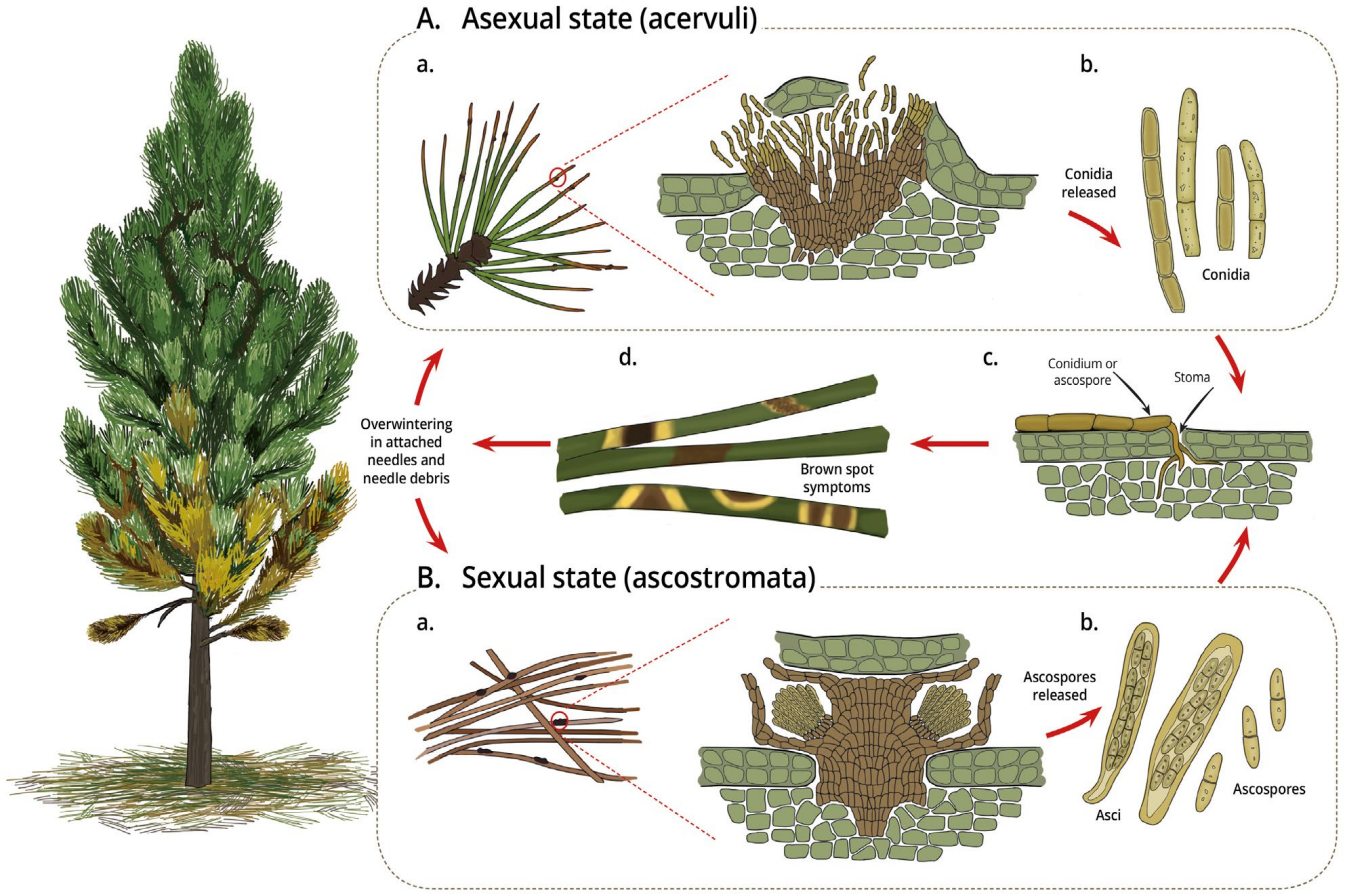
Life cycle of *Lecanosticta acicola* on *Pinus* spp. (A) Asexual state: acervuli (a) develop on attached needles and needle debris and release conidia (b). Infection occurs through the stomata of new season needles (c), resulting in brown spot symptoms (d). (B) Sexual state: ascostromata develop on dead needles associated with previous season infections (a) and release ascospores in spring (b). Infection occurs through the stomata of new season needles (c), resulting in brown spot symptoms (d).

The BSNB pathogen completes its life cycle (Fig. 1) on pine needles that are shed prematurely. This leads to reduced or stunted growth that can result in significant yield losses (Wakeley, 1970) or tree death. In some cases, pine plantations have been sufficiently damaged that they have needed to be cleared (Huang *et al*., 1995; Lévy, 1996; Markovskaja *et al*., 2011).

*Lecanosticta acicola* has been recorded on 53 different *Pinus* species and hybrids in native and non‐native pine stands in the USA, Canada, several European countries and Asia as well as in Central America and Colombia (Table 1). Due to the severity of the disease, the pathogen has been afforded an A1 quarantine status in Africa, Argentina, Chile, Uruguay, Bahrain, Kazakhstan, Ukraine and Russia, and A2 quarantine status in Europe (https://gd.eppo.int/taxon/SCIRAC/categorization). However, reports of new outbreaks of the disease in various European countries have increased significantly since 2008 (Adamson *et al*., 2015, 2018; Anonymous, 2012; Cleary *et al*., 2019; Hintsteiner *et al*., 2012; Jankovský *et al*., 2009a; Markovskaja *et al*., 2011; Mullett *et al*., 2018; Ortíz de Urbina *et al*., 2017).

**Table 1:** Host and geographical range of *Lecanosticta* species.

Country, region, locality	Year collected	Host	Identification method and additional notes	Identification verified using molecular methods (*)	Reported severity of infections and applied eradication methods	Report references
*Lecanosticta acicola*						
Austria, Lower Austria, Valley of the river Ybbs	1996–2000	*P. mugo, P. sylvestris*	Morphological identifications of the pathogen were performed.		Infected trees were eradicated after which the disease was no longer detected (2001–2002).	Brandstetter and Cech (2003)
Austria, Lower Austria, Hollenstein/Ybbs	2008–2009	*P. sylvestris*	The pathogen was recognized during a forest survey.			Cech and Krehan (2008), Kessler (2009)
Austria, Lower Austria, Hollenstein/Ybbs	2009–2010	*P. mugo* subsp. *mugo, P. mugo* subsp. *uncinata*	Symptoms were observed in a survey.			Kessler and Krehan (2011)
Austria, Lower Austria	1996	*P. mugo*	Fruiting bodies were observed on pine needles.		Isolated occurrence in a garden.	Cech (1997)
Austria, Lower Austria, Hollenstein/Ybbs	1998	*Pinus* sp.	Symptoms were observed in the field.			Brandstetter and Cech (1999)
Austria, Lower Austria	2004	*P. mugo, P. sylvestris*	*TEF 1* sequencing used for identification, both mating types were detected.	*		Janoušek *et al*. (2016)
Austria, Lower Austria	2010	*P. mugo*	*TEF 1* sequencing used for identification, both mating types were detected.	*		Janoušek *et al*. (2016)
Austria, Upper Austria	2010	*P. mugo*	*TEF 1* sequencing used for identification. Mating type 1 was detected.	*		Janoušek *et al*. (2016)
Austria, Upper Austria, Bregenz (Vorarlberg)	2011	*P. mugo* subsp. *mugo*	Symptoms were observed in a survey.			Kessler and Krehan (2011)
Austria, Upper Austria, Gmunden	2011	*P. nigra* var. *nigra, P. mugo* subsp. *mugo*	ITS sequencing used for identification.	*		Hintsteiner *et al*. (2012)
Austria, Upper Austria, Tyrol	2011	*P. mugo* subsp. *uncinata*	Symptoms were observed in a survey.			Kessler and Krehan (2011)
Austria, Upper Austria	2012	*P. nigra*	*TEF 1* sequencing used for identification. Mating type 2 was detected.	*		Janoušek *et al*. (2016)
Austria, Upper Austria, Tyrol	2015	*P. mugo* subsp. *mugo, P. mugo* subsp*. uncinata, P. sylvestris*	The pathogen was detected during a forest survey and confirmed with laboratory tests (method not specified).	*	Detected in area covering more than 60 ha of forest.	EPPO (2015)
Austria, Graz	2016	*P. mugo*	Infected needles were collected by I. Barnes. Isolations were made by I. Barnes and A. van der Nest, and identified by ITS sequencing. Mating type 2 was detected.	*	Trees heavily infected (see Fig. 2A,B).	I. Barnes, FABI, Pretoria, South Africa, personal communication
Austria, Lower Austria	2016	*P. mugo*	Infected needles were collected by T. Cech. Isolations were made by I. Barnes and A. van der Nest, and identified by ITS sequencing. Mating type 2 was detected.	*		I. Barnes, FABI, Pretoria, South Africa, personal communication
Austria, Salzburg	2016	*P. uncinata*	Infected needles were collected by T. Cech. Isolations were made by I. Barnes and A. van der Nest, and identified by ITS sequencing. Mating type 2 was detected.	*		I. Barnes, FABI, Pretoria, South Africa, personal communication
Austria, Upper Austria	2016	*P. mugo*	Infected needles were collected by T. Cech. Isolations were made by I. Barnes and A. van der Nest, and identified by ITS sequencing. Mating type 1 was detected.	*		I. Barnes, FABI, Pretoria, South Africa, personal communication
Belize	1981	*P. caribaea, P. oocarpa*	Morphological identifications were made. Confirmation is needed as molecular identification did not reveal *L. acicola* in Central America (van der Nest *et al*., 2019).			Evans (1984)
Bulgaria, near Sofia	1938	*P. nigra*	The pathogen was identified based on morphological characteristics. However, the conidial descriptions are not typical of *L. acicola* and therefore this record is doubtful and should be verified.			Kovaćevski (1938)
Canada, Manitoba	1965	*P. banksiana, P. contorta* var. *latifolia*	Symptoms were observed in the field and the presence of the pathogen was confirmed with morphological identifications.		50–90% of *P. contorta* var. *latifolia *was infected, 20% of *P. banksiana* was infected.	Laut *et al*. (1966)
Canada, New Brunswick, Quebec and Ontario	2009	*P. strobus*	*L. acicola* was reported to occur with *Canavergella banfieldii* on all trees sampled and confirmed based on morphological characteristics.			Laflamme *et al*. (2010)
Canada, Quebec	2011	*P. strobus, P. mugo*	*TEF 1* sequencing used for identification, both mating types detected.	*		Janoušek *et al*. (2016)
China, Jiangsu	1958	*P. thunbergii*	Identification method not specified.		Insignificant damage was reported.	Ye and Wu (2011)
China, Fujian province	1982–1985	*P. elliottii*	Morphological identifications of the pathogen.			Li *et al*. (1987)
China, Anhui, Fujian, Guangdong, Guangxi, Jiangsu, Jiangxi and Zhejiang provinces	1986	*P. caribaea, P. clausa, P. echinata, P. elliottii*, *P. palustris, P. taeda, P. thunbergii*	Morphological characteristics were used to identify the pathogen.		*P. elliottii, P. taeda* and *P. thunbergii* were severely damaged. *P. caribaea, P. clausa, P. echinata* and *P. palustris* were reported as susceptible.	Li *et al*. (1986), Ye and Wu (2011)
China, Fujie	1988	*P. elliottii*	Morphological characteristics and RAPD analysis were used to identify the pathogen. *TEF 1* sequencing was further used for identification and mating type 2 was detected by Janoušek *et al*. (2016).	*		Huang *et al*. (1995), Janoušek *et al*. (2016)
China, Zhejiang	1991	*P. thunbergii*	Morphological characteristics and RAPD analysis were used to identify the pathogen.	*		Huang *et al*. (1995)
China, Jiangxi	1992	*P. elliottii, P. thunbergii*	Morphological characteristics and RAPD analysis were used to identify the pathogen.	*		Huang *et al*. (1995)
China, Guanxi	1992	*P. caribaea, P. elliottii*	Morphological characteristics and RAPD analysis were used to identify the pathogen.	*		Huang *et al*. (1995)
Colombia, Piedras Blancas and Pereira	1978	*P. radiata, P. elliottii, P. patula*	Identification method not specified.		*P. radiata* severely defoliated but on *P. elliottii* and *P. patula* the pathogen was isolated from cast needles found underneath healthy trees.	Gibson (1980)
Colombia, Albán	1981	*P. radiata*	Morphological identification, sexual and asexual state were identified.		Plantations were severely defoliated.	Evans (1984)
Colombia, Refocosta	2011	*P. caribaea*	Infected needles were collected by C.A. Rodas. Isolations were made by I. Barnes. *TEF 1* sequencing was used for identification and mating type 2 was detected by Janoušek *et al*. (2016).	*		Janoušek *et al*. (2016)
Costa Rica, Alajuela	1980	*P. oocarpa*	Morphological identification of pathogen.			Evans (1984)
Croatia, Dalmatia	1975	*P. halepensis*	Morphological identification of *L. acicola.*		The pathogen is not as aggressive as in the USA on this host and it seems to only be aggressive where dense canopies are present with high air humidity. Copper fungicides were applied.	Milatović (1976)
Croatia, Zadar	Not specified	*P. halepensis*	Forest surveys were conducted. It is not specified in the English abstract whether morphological identifications were performed.		500 ha of *P. halepensis* was heavily infected with the pathogen. Highly infected trees and lower infected branches were cut down and it is reported that the trees recovered.	Glavaš and Margaletić (2001)
Croatia, Zadar	2009	*P. halepensis*	*TEF 1* sequencing was used for identification. Mating type 2 was detected.	*		Janoušek *et al*. (2016)
Croatia, Kožino	2015	*P. halepensis*	*TEF 1* sequencing was used for identification. Mating type 2 was detected.	*		Sadiković *et al*. (2019)
Cuba, Baracoa, Guantánamo, Plateau of Mayarí and Master Saw	1980–1998	*P. caribaea, P. cubensis, P. maestrensis*	Symptom identification and morphological confirmation of the fungus.		Mostly seedlings in nurseries were infected.	Lopéz Castilla *et al*. (2002)
Czech Republic, Southern Bohemia, Červené Blato Nature Reserve	2007	*P. uncinata* subsp*. uliginosa*	Morphological identifications were conducted as well as sequencing of the ITS region. The identity of the pathogen was again confirmed with *TEF 1* sequencing by Janoušek *et al*. (2016). Both mating types were detected.	*	Heavy defoliation was reported in 2007. No control measures were taken as the incidence was reported in a natural nature reserve.	Jankovský *et al*. (2009b), Janoušek *et al*. (2016)
Czech Republic, Southern Bohemia, Soběslav, Borkovická Blata National Nature Reserve	2008	*P. uncinata* subsp*. uliginosa*	Morphological identifications were conducted as well as sequencing of the ITS region. The identity of the pathogen was again confirmed with *TEF 1* sequencing by Janoušek *et al*. (2016). Both mating types were detected.	*	No action was taken as the outbreak was in a natural reserve.	Jankovský *et al*. (2009a), Janoušek *et al*. (2016)
Estonia, Hiiumaa Island and Käravere	2014–2015	*P. mugo*	Symptom identification was confirmed with conventional PCR directly from pine needles. *Lecanosticta acicola* was isolated from the needles and confirmed with ITS sequencing. Both mating types were detected.	*		Adamson *et al*. (2015)
Estonia, Tallinn Botanical Garden	2006–2008	*P. ponderosa*	Material of *Dothistroma* was collected and isolated but in culture it was determined to be *L. acicola* based on culture morphology. The *TEF 1* sequences were later determined for representative isolates and mating type 2 was detected (Janoušek *et al*., 2016).	*		Drenkhan and Hanso (2009), Janoušek *et al*. (2016)
Estonia, Tallinn Botanical Garden	2010–2013	*P. mugo, P. mugo* var. *pumilio, P. ponderosa, P. uncinata*	Symptom identification was confirmed with conventional PCR directly from pine needles. *Lecanosticta acicola* was isolated from the needles and confirmed with ITS sequencing. Mating type 1 was detected.	*		Adamson *et al*. (2015)
Estonia, Tartu county	2016	*P. sylvestris, P. mugo, Pinus* × *rhaetica*	Visual symptom identification was confirmed with conventional PCR and selected isolates were identified using an ITS sequencing PCR. Both mating types were detected.	*		Adamson *et al*. (2018)
Estonia, Tori and Vasula	2012, 2013	*P. mugo*	Symptom identification was confirmed with conventional PCR directly from pine needles. *Lecanosticta acicola* was isolated from the needles and confirmed with ITS sequencing.	*		Adamson *et al*. (2015)
France, South‐West, Aquitaine and western Pyrénées	1993	*P. attenuata* × *P. radiata*	In field observations were made.		Severe tree mortality was observed. French authorities implemented eradication measures and destroyed 127 ha of trees.	Lévy (1996)
France, Gironde	1995	*P. muricata*	*TEF 1* and *BT 2* sequencing used for identification, mating type 1 detected.	*		Ioos *et al*. (2010), Janoušek *et al*. (2016)
France, Landes	1995	*P. attenuata* × *P. radiata*	*TEF 1* and *BT 2* sequencing used for identification, mating type 2 detected.	*		Ioos *et al*. (2010), Janoušek *et al*. (2016)
France, Pyrénées‐Atlantiques	1995	*P. radiata*	*TEF 1* and *BT 2* sequencing used for identification.	*		Ioos *et al*. (2010)
France, Ariège	2009	*P. sylvestris*	Forest surveys were conducted.		More than 50% of the trees were affected.	Alvère *et al*. (2010)
France, Tarn‐et‐Garonne	2009	*P. nigra* var*. laricio*	Forest surveys were conducted.		The trees were moderately affected.	Alvère *et al*. (2010)
France, Pyrénées‐Atlantiques	2012	*P. radiata*	*TEF 1* sequencing used for identification, both mating types were detected.	*		Janoušek *et al*. (2016)
Germany, Bavaria	1994	*P. mugo*	The pathogen was identified based on morphological characteristics.			Pehl (1995)
Germany, Bavaria	1994, 2000, 2010, 2011	*P. mugo*	*TEF 1* sequencing used for identification, both mating types were detected.	*		Janoušek *et al*. (2016)
Germany, Bavaria, Munich Botanical gardens	2018	*P. mugo*	Collected by I. Barnes. The identity was confirmed by ITS sequencing. *Dothistroma septosporum* was also present.	*		I. Barnes, FABI, Pretoria, South Africa, personal communication
Guatemala, El Progreso	1983	*P. oocarpa*	Morphological identification methods were used. As *L. acicola* was not identified in Central America using molecular identification techniques (van der Nest *et al*., 2019), this report will need to be verified.			Evans (1984)
Honduras	1980–1983	*P. caribaea, P. maximinoi, P. oocarpa, P. tecunumanii, *	Morphological identification methods were used. As *L. acicola* was not identified in Central America using molecular identification techniques (van der Nest *et al*., 2019), this report will need to be verified.			Evans (1984)
Ireland, Wexford county	2016	*P. mugo, P. sylvestris*	ITS sequencing was used for identification purposes. Mating type 1 was detected.	*		Mullett *et al*. (2018)
Italy, Brescia	1997	*P. mugo*	Symptoms were noted in the botanical garden and the presence of the pathogen was confirmed with morphological identifications.		Extensive necrosis and crown defoliation were observed in all 12 of the *P. mugo* trees present in the botanical garden.	La Porta and Capretti (2000)
Italy, Brescia	2008	*P. mugo*	*TEF 1* sequencing used for identification and mating type 1 detected.	*		Janoušek *et al*. (2016)
Japan, Shimane Prefecture (Honshu)	1996	*P. thunbergii, P. densiflora* (tested in controlled environment)	The pathogen was morphologically identified.		*P. thunbergii* was severely infected. Inoculation trials on this host as well as *P. densiflora* also revealed that *P. densiflora* is susceptible although it was not reported in the host's natural environment.	Suto and Ougi (1998)
Japan, Shimane	2010	*P. thunbergii*	*TEF 1* sequencing used for identification, mating type 2 was detected.	*		Janoušek *et al*. (2016)
Latvia, Salaspils	2012	*P. pumila*	Morphological identification. Later it was confirmed with PCR‐based methods.	*	Eradication measures were taken.	EPPO (2012a)
Latvia, Salaspils	2016	*P. mugo*	Identification was done by ITS sequencing. Mating type 1 was detected.	*		Mullett *et al*. (2018)
Lithuania, Curonian Spit, Smiltynė Forest District	2009	*P. mugo*	Morphological characteristics as well as ITS sequencing and ITS‐RFLP was used to identify the pathogen. This material was again examined by Janoušek *et al*. (2016) and the identity confirmed with *TEF 1*. Mating type 1 was detected.	*	A monitoring programme was initiated and infected trees felled and burned.	Markovskaja *et al*. (2011), Janoušek *et al*. (2016)
Lithuania, Curonian Spit, Smiltynė Forest District and Juodkrantė Forest District	2010	*P. mugo*	Morphological characteristics as well as ITS sequencing and ITS‐RFLP was used to identify the pathogen.	*	A monitoring programme was initiated and infected trees felled and burned.	Markovskaja *et al*. (2011)
Lithuania, Curonian Spit, near Juodkrante	2012	*P. mugo, P. sylvestris*	Morphological identifications and PCR‐based methods.	*	Phytosanitary methods were implemented.	EPPO (2012b)
Lithuania, Curonian Spit, Smiltyne Smiltynė Forest District and Juodkrantė Forest District	2014	*P. mugo*	Infected needles were collected by S. Markovskaja. Isolations were made by A. van der Nest. A multigene phylogenetic approach was used to determine the identity of the isolates.	*		van der Nest *et al*. (2019)
Mexico, Puebla	1983	*P. patula*	Morphological identification.			Evans (1984)
Mexico	2000	*P. ayacahuite, P. cembroides, P. halepensis*	Morphological characteristics were examined.		High disease severity was reported on *P. halepensis.*	Marmolejo (2000)
Mexico, Nuevo León	2010, 2011	*P. halepensis*	*TEF 1* sequencing used for identification, both mating types detected. KJ938447–KJ938449 were later identified as *L. variabilis* (van der Nest *et al*., 2019) and the remaining isolates are part of *L. acicola* lineage 3.	*		Janoušek *et al*. (2016)
Nicaragua	1981–1983	*P. caribaea, P. maximinoi, P. oocarpa, P. tecunumanii*	Morphological identification methods were used. As *L. acicola* was not identified in Central America using molecular identification techniques (van der Nest *et al*., 2019), this report will need to be verified. Both the sexual and asexual state was observed.			Evans (1984)
Portugal, Minho	2016	*P. radiata*	Identification was done by ITS sequencing. Mating type 1 was detected.	*		Mullett *et al*. (2018)
Romania, Vrancea	2017	*Pinus* sp.	The pathogen was detected during a forest survey in a 30‐year‐old plantation.		Eradication reported to be under way in the 19‐hectare forest.	EPPO (2018)
Russia, Krasnodar region, Sochi	2016	*P. mugo* subsp. *mugo, P. thunbergii*	Identification was done by ITS sequencing. Mating type 2 was detected.	*		Mullett *et al*. (2018)
Slovenia, Bled	2008–2009	*P. mugo, P. sylvestris*	Morphological identifications. The identity of isolates on *P. mugo* were confirmed with *TEF 1* sequencing and mating type 2 was detected (Janoušek *et al*., 2016; Sadiković *et al*., 2019).	*	All affected trees were eradicated.	Jurc and Jurc (2010), Janoušek *et al*. (2016), Sadiković *et al*. (2019)
Slovenia, Čatež	2015	*P. mugo*	*TEF 1* sequencing was used for identification. Mating type 1 was detected.	*		Sadiković *et al*. (2019)
Slovenia, Ljubljana	2008–2009	*P. mugo, P. sylvestris*	Morphological identifications. The identity of isolates from *P. mugo* were confirmed with *TEF 1* sequencing by Sadiković *et al*. (2019).	*	All affected trees were eradicated.	Jurc and Jurc (2010), Sadiković *et al*. (2019)
Slovenia, Ljubljana	2013	*P. mugo*	*TEF 1* sequencing was used for identification. Mating type 1 was detected.	*		Sadiković *et al*. (2019)
Slovenia, Tolmin	2016	*P. nigra*	*TEF 1* sequencing was used for identification. Mating type 1 was detected.	*		Sadiković *et al*. (2019)
Slovenia, Trenta	2014–2015	*P. mugo*	*TEF 1* sequencing was used for identification. Mating type 2 was detected.	*		Sadiković *et al*. (2019)
South Korea, Naju	2010–2011	*P. thunbergii*	*L. acicola* symptoms were observed and confirmed with ITS sequencing. *TEF 1* sequencing was used for identification by Janoušek *et al*. (2016) and mating type 2 was detected.	*	Low incidence, less than 1%.	Janoušek *et al*. (2016), Seo *et al*. (2012)
Spain	1942	*P. radiata*	Probably oldest official report of *L. acicola* in Europe based on morphological identification.			Martínez (1942)
Spain, Cantabria	2012	*P. radiata*	*TEF 1* sequencing used for identification, mating type 2 was detected.	*		Janoušek *et al*. (2016)
Spain, Spanish Atlantic climate region	2015	*P. nigra, P. radiata*	Sequenced directly from needles using conventional PCR (Ioos *et al*., 2010). Both mating types were detected.	*	*Lecanosticta acicola* was detected on 44.7% of trees that were surveyed.	Ortíz de Urbina *et al*. (2017)
Sweden	2017	*P. mugo *‘Hesse’	Morphological identification and ITS sequencing.	*	Single tree in arboretum that was severely affected.	Cleary *et al*. (2019)
Switzerland, Zollikon	1995	*P. mugo, P. uncinata*	Morphological identification of the pathogen.		Control measures were initiated in accordance with the phytosanitary policy of the EPPO.	Holdenrieder and Sieber (1995)
Switzerland, Canton St Gallen	1999	*P. mugo*	*TEF 1* sequencing used for identification.	*		Janoušek *et al*. (2016)
Switzerland, Canton Zug	2009	*P. mugo*	Symptoms were observed in the field. Later, *TEF 1* sequencing was used to confirm identification (Janoušek *et al*., 2016). Mating type 1 was detected.	*		Angst (2011), Janoušek *et al*. (2016)
Switzerland, Zürich	2009	*P. mugo*	Symptoms were observed in the field. Later, *TEF 1* sequencing was used to confirm identification (Janoušek *et al*., 2016). Mating type 1 was detected.	*		Angst (2011), Janoušek *et al*. (2016)
Switzerland, Bern and Zürich	2017	*P. mugo*	Detection with qPCR and a conventional PCR directly from pine needles.	*		Schneider *et al*. (2019)
Switzerland, Schwyz	2017	*P. sylvestris*	Detection with qPCR and a conventional PCR directly from pine needles.	*		Schneider *et al*. (2019)
USA, Alabama	1929	*P. palustris*	Hedgcock reported on collections of the pathogen at the office of Forest Pathology at Washington, D.C. and the Mycological collections of the US Department of Agriculture.			Hedgcock (1929)
USA, Alabama	1944	*P. echinata, P. palustris, P. taeda*	Siggers reported *Lecanosticta* isolates that are in the collections in the Division of Forest Pathology in Louisiana and Maryland, USA. These reports should be verified.			Siggers (1944)
USA, Alabama	1948–1967	*P. palustris*	Symptoms were observed annually on seedlings and the proportion of seedlings affected were recorded.		In a 4‐year study, 78% or more seedlings were infected yearly with *L. acicola.*	Boyer (1972)
USA, Arkansas	1929	*P. taeda*	Hedgcock reported on collections of the pathogen at the office of Forest Pathology at Washington, D.C. and the Mycological collections of the US Department of Agriculture.			Hedgcock (1929)
USA, Arkansas	1944	*P. taeda*	Siggers reported *Lecanosticta* isolates that are in the collections in the Division of Forest Pathology in Louisiana and Maryland, USA. These reports should be verified.			Siggers (1944)
USA, Arkansas	1967–1971	*P. sylvestris*	Symptoms were observed in the field and the proportion of needles affected were noted. In some cases, microscopic examinations of conidia were used for identification.			Skilling and Nicholls (1974)
USA, Florida	1929	*P. caribaea, P. glabra, P. palustris, P. taeda*	Hedgcock reported on collections of the pathogen at the office of Forest Pathology at Washington, D.C. and the Mycological collections of the US Department of Agriculture.			Hedgcock (1929)
USA, Florida	1944	*P. attenuata, P. caribaea, P. coulteri, P. jeffreyi, P. glabra, P. halepensis, P. latifolia, P. muricata, P. palustris, P. pinaster, P. pinea, P. ponderosa* var*. scopulorum, * * P. radiata, P. thunbergii*	Siggers reported on *Lecanosticta* isolates that are in the collections in the Division of Forest Pathology in Louisiana and Maryland, USA. These reports should be verified.			Siggers (1944)
USA, Georgia	1929	*P. palustris, P. taeda, P. virginiana*	Hedgcock reported on collections of the pathogen at the office of Forest Pathology at Washington, D.C. and the Mycological collections of the US Department of Agriculture.			Hedgcock (1929)
USA, Georgia	1944	*P. caribaea, P. palustris, P. taeda, P. virginiana*	Siggers reported *Lecanosticta* isolates that are in the collections in the Division of Forest Pathology in Louisiana and Maryland, USA. These reports should be verified.			Siggers (1944)
USA, Idaho	1929	*P. ponderosa*	Hedgcock reported on collections of the pathogen at the office of Forest Pathology at Washington, D.C. and the Mycological collections of the US Department of Agriculture. According to Siggers (1944) the identification was based on characteristics that do not fit *Lecanosticta* and therefore this record should be verified.			Hedgcock (1929)
USA, Iowa	1967–1971	*P. sylvestris*	Symptoms were observed in the field and the proportion of needles affected were noted. In some cases, microscopic examinations of conidia were used for identification.			Skilling and Nicholls (1974)
USA, Kansas	1929	*P. nigra* var. *austriaca*	Hedgcock reported on collections of the pathogen at the office of Forest Pathology at Washington, D.C. and the Mycological collections of the US Department of Agriculture. According to Siggers (1944) the identification was based on characteristics that do not fit *Lecanosticta* and therefore this record should be verified.			Hedgcock (1929)
USA, Kansas	1951	*P. nigra, P. ponderosa*	Reports in the field and mycological identification.			Rogerson (1953)
USA, Kansas	1967–1971	*P. sylvestris*	Symptoms were observed in the field and the proportion of needles affected were noted. In some cases, microscopic examinations of conidia were used for identification.			Skilling and Nicholls (1974)
USA, Kentucky	1929	*P. nigra* var. *austriaca*	Hedgcock reported on collections of the pathogen at the office of Forest Pathology at Washington, D.C. and the Mycological collections of the US Department of Agriculture. According to Siggers (1944) the identification was based on characteristics that do not fit *Lecanosticta* and therefore this record should be verified.			Hedgcock (1929)
USA, Kentucky	1967–1971	*P. sylvestris*	Symptoms were observed in the field and the proportion of needles affected were noted. In some cases, microscopic examinations of conidia were used for identification.			Skilling and Nicholls (1974)
USA, Louisiana	1929	*P. palustris, Pinus * × * sondereggeri, P. taeda*	Hedgcock reported on collections of the pathogen at the office of Forest Pathology at Washington, D.C. and the Mycological collections of the US Department of Agriculture.			Hedgcock (1929)
USA, Louisiana	1929–1930, 1960	*P. palustris*	Symptoms were observed and the proportion of seedlings affected were recorded at 4–5 years of age and again at 30 years.		Most of the trees were affected.	Wakeley (1970)
USA, Louisiana	1944	*P. attenuata, P. caribaea, P. contorta* var*. latifolia, P. echinata, P. nigra* var*. laricio, P. palustris, P. pinaster, P. ponderosa* var*. scopulorum, P. radiata, P. rigida, P. serotina, P. sabiniana, Pinus *× *sondereggeri, P. taeda*	Siggers reported on *Lecanosticta* isolates that are in the collections in the Division of Forest Pathology in Louisiana and Maryland, USA. These reports should be verified.			Siggers (1944)
USA, Maine	2011	*P. strobus*	Isolates were collected and morphologically identified in a survey. These isolates were later identified with *TEF 1* sequencing and both mating types were detected.	*		Munck *et al*. (2012), Janoušek *et al*. (2016)
USA, Maine	2011–2012	*P. strobus*	*Lecanosticta acicola* was identified as part of a complex of pathogens that cause white pine needle damage (WPND). Morphological identifications and selected ITS PCR sequencing was performed to confirm the presence of *L. acicola*.	*	It was observed that affected trees were defoliated annually.	Broders *et al*. (2015)
USA, Michigan	2016	*P. sylvestris*	*TEF 1* sequencing used for identification, mating type 2 was detected.	*		Janoušek *et al*. (2016)
USA, Minnesota	1967–1971	*P. sylvestris*	Symptoms were observed in the field and the proportion of needles affected were noted. In some cases, microscopic examinations of conidia were used for identification.			Skilling and Nicholls (1974)
USA, Minnesota and Wisconsin	1970–1972	*P. banksiana, P. glauca, P. nigra, P. palustris, P. resinosa, P. strobus, P. sylvestris, Picea glauca*	Symptoms were observed in the field and the proportion of needles affected were noted.		These species were tested for susceptibility in a field trial by planting the hosts underneath heavily infected *P. sylvestris*. Four varieties of *P. sylvestris,* as well as *P. nigra* and *P. resinosa,* were the most susceptible. *P. strobus* was moderately resistant. *P. banksiana* was the most resistant. Less than 1% of *Picea glauca* was infected.	Skilling and Nicholls (1974)
USA, Mississippi	1929	*P. caribaea, P. palustris, P. taeda*	Hedgcock reported on collections of the pathogen at the office of Forest Pathology at Washington, D.C. and the Mycological collections of the US Department of Agriculture.			Hedgcock (1929)
USA, Mississippi	1944	*P. caribaea, P. palustris, P. pinaster, P. taeda, P. thunbergii*	Siggers reported on *Lecanosticta* isolates that are in the collections in the Division of Forest Pathology in Louisiana and Maryland, USA. These reports should be verified.			Siggers (1944)
USA, Mississippi	1952–1953	*P. palustris*	Microscopic identification. Both the sexual and asexual states were observed.			Henry (1954)
USA, Mississippi	1966–1967	*P. palustris*	Morphological identifications. Both the sexual state and asexual state were observed throughout the year on infected *P. palustris.*			Kais (1971)
USA, Mississippi	2012	*P. palustris, P. taeda*	*TEF 1* sequencing used for identification, both mating types detected.	*		Janoušek *et al*. (2016)
USA, Missouri	1929	*P. nigra* var. *austriaca*	Hedgcock reported on collections of the pathogen at the office of Forest Pathology at Washington, D.C. and the Mycological collections of the US Department of Agriculture. According to Siggers (1944) the identification was based on characteristics that do not fit *Lecanosticta* and therefore this record should be verified.			Hedgcock (1929)
USA, Missouri	1947–1949	*P. ponderosa*	Symptoms were observed in the field and morphological identifications were made. Both the sexual state and asexual state were observed.		All trees were affected. Excessive needle defoliation and in some cases tree mortality was observed.	Luttrell (1949)
USA, Missouri	1967–1971	*P. sylvestris*	Symptoms were observed in the field and the proportion of needles affected were noted. In some cases, microscopic examinations of conidia were used for identification.			Skilling and Nicholls (1974)
USA, New England	2016	*P. strobus*	Severe needle browning was observed and *L. acicola* was identified as part of a complex of species causing premature defoliation. This is possibly WPND although it was not defined as such.			Brazee (2016)
USA, New Hampshire	2011	*P. strobus*	Isolates were collected and morphologically identified in a survey. These isolates were later identified with *TEF 1* sequencing and mating type 1 was detected.	*		Munck *et al*. (2012), Janoušek *et al*. (2016)
USA, New Hampshire	2011–2012	*P. strobus*	*Lecanosticta acicola* was identified as part of a complex of pathogens that cause WPND. Morphological identifications and selected ITS PCR sequencing confirmed the presence of *L. acicola.*	*	It was observed that affected trees were defoliated annually.	Broders *et al*. (2015)
USA, New York	1976	*P. mugo*	*Lecanosticta acicola* was identified with morphological methods and brown spot needle blight symptoms confirmed on trees. Specimens are in the Cornell University Plant Pathology Herbarium.			Sinclair and Hudler (1980)
USA, North Carolina	1929	*P. echinata, P. palustris, P. rigida, P. taeda, P. virginiana*	Hedgcock reported on collections of the pathogen at the office of Forest Pathology at Washington, D.C. and the Mycological collections of the US Department of Agriculture.			Hedgcock (1929)
USA, North Carolina	1944	*P. palustris, P. rigida, P. strobus, P. taeda, P. virginiana*	Siggers reported on *Lecanosticta* isolates that are in the collections in the Division of Forest Pathology in Louisiana and Maryland, USA. These reports should be verified.			Siggers (1944)
USA, North Carolina	1957, 1958	*P. strobus*	Morphological identifications of *L. acicola*.			Boyce (1959)
USA, Ohio	1944	*P. contorta* var*. latifolia, P. coulteri, P. * * jeffreyi*	Siggers reported on *Lecanosticta* isolates that are in the collections in the Division of Forest Pathology in Louisiana and Maryland, USA. These reports should be verified.			Siggers (1944)
USA, Oregon	1929	*P. attenuata*	Hedgcock reported on collections of the pathogen at the office of Forest Pathology at Washington, D.C. and the Mycological collections of the US Department of Agriculture.			Hedgcock (1929)
USA, Oregon	1944	*P. attenuata*	Siggers reported on *Lecanosticta* isolates that are in the collections in the Division of Forest Pathology in Louisiana and Maryland, USA. These reports should be verified.			Siggers (1944)
USA, Pennsylvania	1929	*P. rigida*	Hedgcock reported on collections of the pathogen at the office of Forest Pathology at Washington, D.C. and the Mycological collections of the US Department of Agriculture.			Hedgcock (1929)
USA, Pennsylvania	1987–1989	*P. strobus*	Morphological identifications were done.			Stanosz (1990)
USA, South Carolina	1876	*P. echinata* (*P. variabilis*)	Morphological description of *Cryptosporium acicolum.*			de Thümen (1878)
USA, South Carolina	1929	*P. caribaea, P. echinata, P. palustris, P. serotina, P. taeda*	Hedgcock reported on collections of the pathogen at the office of Forest Pathology at Washington, D.C. and the Mycological collections of the US Department of Agriculture.			Hedgcock (1929)
USA, South Carolina	1944	*P. caribaea, P. palustris, P. taeda*	Siggers reported on *Lecanosticta* isolates that are in the collections in the Division of Forest Pathology in Louisiana and Maryland, USA. These reports should be verified.			Siggers (1944)
USA, Tennessee	1929	*P. rigida, P. taeda*	Hedgcock reported on collections of the pathogen at the office of Forest Pathology at Washington, D.C. and the Mycological collections of the US Department of Agriculture.			Hedgcock (1929)
USA, Tennessee	1944	*P. palustris, P. ponderosa* var*. scopulorum, P. rigida, P. taeda*	Siggers reported on *Lecanosticta* isolates that are in the collections in the Division of Forest Pathology in Louisiana and Maryland, USA. These reports should be verified.			Siggers (1944)
USA, Texas	1929	*P. palustris, P. taeda*	Hedgcock reported on collections of the pathogen at the office of Forest Pathology at Washington, D.C. and the Mycological collections of the US Department of Agriculture.			Hedgcock (1929)
USA, Texas	1929	*P. palustris, P. taeda*	Symptoms were observed on trees inside and surrounding the nurseries.		Low severity recorded. Nursery beds were sprayed with Bordeaux 4‐4‐50 with good results.	Webster (1930)
USA, Texas	1944	*P. caribaea, P. palustris, P. pinaster, P. taeda*	Siggers reported on *Lecanosticta* isolates that are in the collections in the Division of Forest Pathology in Louisiana and Maryland, USA. These reports should be verified.			Siggers (1944)
USA, Vermont	2008	*P. mugo, P. resinosa, P. sylvestris, P. strobus*	Forest surveys were conducted and the pathogen identified based on symptomology.			Gibbs and Sinclair (2008)
USA, Vermont	2011	*P. strobus*	Isolates were collected and morphologically identified in a survey. These isolates were later identified with *TEF 1* sequencing and both mating types were detected.	*		Munck *et al*. (2012), Janoušek *et al*. (2016)
USA, Vermont	2011–2012	*P. strobus*	*Lecanosticta acicola* was identified as part of a complex of pathogens that cause WPND. Morphological identifications and selected ITS PCR sequencing confirmed the presence of *L. acicola.*	*	It was observed that affected trees were defoliated annually.	Broders *et al*. (2015)
USA, Virginia	1929	*P. rigida*	Hedgcock reported on collections of the pathogen at the office of Forest Pathology at Washington, D.C. and the Mycological collections of the US Department of Agriculture.			Hedgcock (1929)
USA, Wisconsin	1966–1970	*P. sylvestris*	A forest survey was conducted and symptoms of *L. acicola* was observed.		Approximately 3000 acres in 55 plantations were severely infected. Short leaf French and Spanish *P. sylvestris* were severely affected. Long leaf *P. sylvestris* varieties were reported as resistant.	Prey and Morse (1971)
USA, Wisconsin	1967–1971	*P. sylvestris*	Symptoms were observed in the field and the proportion of needles affected were noted. In some cases, microscopic examinations of conidia were used for identification.			Skilling and Nicholls (1974)
USA, Wisconsin	1970	*P. resinosa*	Symptoms were observed in the field and morphological identifications were made.		After the pathogen was observed in pine stands, an inoculation trial revealed that *P. resinosa* is highly susceptible to *L. acicola*.	Nicholls and Hudler (1972)
USA, Wisconsin	2010	*P. sylvestris*	*TEF 1* sequencing used for identification, mating type 2 was detected.	*		Janoušek *et al*. (2016)
*Lecanosticta brevispora*						
Guatemala, Alta Verapaz, Santa Cruz Verapaz, near Tactíc	2010	*P. oocarpa*	Multigene phylogenetic analysis.	*		van der Nest *et al*. (2019)
Guatemala, Chimaltenango, Tecpán, Finca La Esperanza	2010	*P. pseudostrobus*	Multigene phylogenetic analysis.	*		van der Nest *et al*. (2019)
Guatemala, Lugar, La Soledad, Jalapa site II	2012	*P. oocarpa*	Multigene phylogenetic analysis.	*		van der Nest *et al*. (2019)
Honduras	2010	*P. oocarpa*	Multigene phylogenetic analysis.	*		van der Nest *et al*. (2019)
Mexico	2000	*Pinus* sp.	Multigene phylogenetic analysis.	*		Quaedvlieg *et al*. (2012)
*Lecanosticta gloeospora*						
Mexico, Nuevo León, Iturbide‐Galeana	1983	*P. pseudostrobus*	Morphological identification. The type was later sequenced using multiple genes (van der Nest *et al*., 2019).	*		Evans (1984), Marmolejo (2000), van der Nest *et al*. (2019)
*Lecanosticta guatemalensis*						
Guatemala, Baja Verapaz	1983	*P. oocarpa*	The type culture was previously identified as *L. acicola* based on morphological characteristics (Evans, 1984). Multigene phylogenetic analysis revealed it as a new species, *L. guatemalensis.*	*		Quaedvlieg *et al*. (2012), van der Nest *et al*. (2019)
Guatemala, Alta Verapaz, Santa Cruz Verapaz, near Tactíc	2010	*P. oocarpa*	Multigene phylogenetic analysis.	*	Very low.	van der Nest *et al*. (2019)
Guatemala, Chiquimula	2011	*P. oocarpa*	Multigene phylogenetic analysis.	*	Very low.	van der Nest *et al*. (2019)
Guatemala, Jalapa, Finca Forestal Soledad	2012	*P. oocarpa*	Multigene phylogenetic analysis.	*	Very low.	van der Nest *et al*. (2019)
Guatemala, Coban, San Juan Chamelco	2012	*P. oocarpa*	Multigene phylogenetic analysis.	*	Very low.	van der Nest *et al*. (2019)
Nicaragua	1982	*P. tecunumanii*	This isolate was previously identified as *L. acicola* based on morphological characteristics (Evans, 1984). Multigene phylogenetic analysis revealed it to be *L. guatemalensis.*	*		van der Nest *et al*. (2019)
Nicaragua, Matagalpa	2010	*P. oocarpa*	Multigene phylogenetic analysis.	*	Very low.	van der Nest *et al*. (2019)
Honduras, Yoro	1981	*P. caribaea, P. oocarpa*	These isolates were previously identified as *L. acicola* based on morphological characteristics (Evans, 1984). Multigene phylogenetic analysis revealed it to be *L. guatemalensis.*	*		van der Nest *et al*. (2019)
*Lecanosticta jani*						
Guatemala, Alta Verapaz, Santa Cruz Verapaz, near Tactíc	2010	*P. oocarpa*	Multigene phylogenetic analysis.	*	Very low.	van der Nest *et al*. (2019)
Guatemala, Chiquimula	2010	*P. oocarpa*	Multigene phylogenetic analysis.	*	Very low.	van der Nest *et al*. (2019)
Guatemala, Jalapa, Finca Forestal Soledad	2012	*P. maximinoi*	Multigene phylogenetic analysis.	*	Very low.	van der Nest *et al*. (2019)
Guatemala, Jalapa, Finca La Soledad, Mataquescuintla	2012	*P. tecunumanii*	Multigene phylogenetic analysis.	*	Very low.	van der Nest *et al*. (2019)
Nicaragua, Matagalpa	2010	*P. oocarpa*	Multigene phylogenetic analysis.	*	Very low.	van der Nest *et al*. (2019)
*Lecanosticta pharomachri*						
Guatemala, Baja Verapaz, San Jerónimo, Salamá	2012	*P. tecunumanii*	Multigene phylogenetic analysis.	*	Very low.	van der Nest *et al*. (2019)
Guatemala, Jalapa, Finca La Soledad, Mataquescuintla	2010–2012	*P. oocarpa*	Multigene phylogenetic analysis.	*	Very low.	van der Nest *et al*. (2019)
Honduras	2010	*P. oocarpa*	Multigene phylogenetic analysis.	*	Very low.	van der Nest *et al*. (2019)
*Lecanosticta tecunumanii*						
Guatemala, Baja Verapaz, San Jerónimo, Salamá	2012	*P. tecunumanii*	Multigene phylogenetic analysis.	*	Very low.	van der Nest *et al*. (2019)
*Lecanosticta variabilis*						
Guatemala, Alta Verapaz, Santa Cruz Verapaz, near Tactíc	2010	*P. oocarpa*	Multigene phylogenetic analysis. Both mating types were present (Janoušek *et al*., 2016).	*	Very low.	van der Nest *et al*. (2019)
Guatemala, Jalapa, Finca Forestal Soledad	2012	*P. maximinoi*	Multigene phylogenetic analysis.	*	Very low.	van der Nest *et al*. (2019)
Honduras, Santa Barbara, Lago de Yojoa	1984	*P. caribaea*	This isolate was previously identified as *L. acicola* in a morphological study by Evans (1984). A multigene phylogenetic analysis indicated that this is a new species, *L. tecunumanii.*	*	Very low.	Evans (1984), van der Nest *et al*. (2019)
Mexico	2000	*Pinus* sp.	Multigene phylogenetic analysis.	*		van der Nest *et al*. (2019)
Mexico	2010	*P. arizonica* var*. stormiae, P. halepensis*	Multigene phylogenetic analysis. The isolates were previously identified as *L. acicola* (Janoušek *et al*., 2016) and both mating types were detected.	*		van der Nest *et al*. (2019)

Quarantine measures rely on accurately identifying the presence of pathogens on symptomatic tissues. This is complicated in the case of *L. acicola* where the symptoms of BSNB closely resemble those of Dothistroma needle blight (DNB). DNB is caused by two species: *Dothistroma septosporum* and *D. pini* (Barnes *et al*., 2016). Due to their similar symptoms, field diagnoses of the causal agent based on symptoms and/or on morphology alone have commonly been incorrect (Shishkina and Tsanava, 1967; Siggers, 1944; Thyr and Shaw, 1964). Consequently, past reports of *L. acicola* based only on morphological descriptions and symptoms must be treated with caution and verified using molecular identification techniques (van der Nest *et al*., 2019).

*Lecanosticta acicola* has been well‐known in the southeastern USA since the early 1900s, but is rapidly spreading in northern parts of the USA, Canada and in some parts of Europe (Broders *et al*., 2015). Its complete host range is not known but appears to be expanding (Mullett *et al*., 2018). A recent taxonomic re‐evaluation of isolates previously identified as *L. acicola*, applying phylogenetic analyses based on DNA sequences, has led to various isolates being recognized as distinct species (Quaedvlieg *et al*., 2012; van der Nest *et al*., 2019). This and a number of recent publications (Adamson *et al*., 2018; Cleary *et al*., 2019; Mullett *et al*., 2018; Ondrušková *et al*., 2018; Ortíz de Urbina *et al*., 2017; Sadiković *et al*., 2019; Schneider *et al*., 2019; Wyka *et al*., 2017) justifies the need for a review of current knowledge regarding BSNB and the *Lecanosticta* species that cause this disease. This is the first review of the topic to be presented in 75 years subsequent to that of Siggers (1944).

## *Lecanosticta* Species

The genus *Lecanosticta,* which includes nine species with the type species being *L. acicola* (previously known as *Mycosphaerella dearnessii,* Table 2)*,* is characterized by stromata and septate, pigmented conidia. The genus was erected by Sydow and Petrak in 1922 (Sydow and Petrak, 1922). The taxonomic history and nomenclature of *Lecanosticta acicola* has been succinctly presented previously (Evans, 1984; Siggers, 1944) and is summarized and updated in Table 2.

**Table 2:** A summarized history of the taxonomy and nomenclature of the genus *Lecanosticta*.

Year	Species epithet	Reference	Sexual state reported	Country, location	Host	Description	Notes
*Lecanosticta acicola*							
1878	*Cryptosporium acicolum Thüm *	de Thümen (1878)	Asexual	USA, South Carolina, Aiken	*Pinus echinata* (*P. variabilis*)		*P. variabilis* is a synonym of *P. echinata.* In Wolf and Barbour (1941), it was mentioned that it was in fact on *P. caribaea* and that the host was previously incorrectly identified.
1884	*Septoria acicola* (Thüm) Sacc	Saccardo (1884)	Asexual	USA, Carolina, Aiken	*P. variabilis*	Saccardo moved *C. acicolum* to *Septoria* due to the characteristic septate conidia.	
1922	*Lecanosticta pini*	Sydow and Petrak (1922)	Asexual	USA, Arkansas and Oregon	*P. taeda* and *P. palustris* in Arkansas and *P. attenuata* in Oregon	The genus *Lecanosticta* was erected to accommodate *L. pini,* a fungus with erumpent stromata and pigmented conidia.	
1924	*Lecanosticta acicola*	Sydow and Petrak (1924)	Asexual	USA	–	The authors recognized that *L. pini* was *C. acicolum.* The genus was retained and the name *L. acicola* was proposed as the valid name.	
1926	*Oligostroma acicola*	Dearness (1926, 1928)	Sexual	USA, Florida, Silver Springs	*P. palustris*	The sexual state of *L. acicola* was isolated from old pine needles from which the asexual state was previously isolated. In 1928, it was proposed that the asexual state *Septoria acicola* fits better in the genus *Cryptosporium* and that *Oligostroma acicola* could be *Cryptosporium acicolum*’s sexual state (Dearness, 1928).	
1939	*Schirria acicola*	Siggers (1939)	Sexual	USA, Arkansas, Florida, Georgia, Louisiana, North Carolina, Texas	*P. palustris, P. taeda*, *P. thunbergii*	Ascospores as well as conidia were plated onto media and morphologically examined to come to the conclusion that the sexual and asexual state are connected. *Oligostroma acicola* was changed to *Schirria acicola* as erumpent acervuli were observed, characteristic of *Schirria*.	
1941	*Systremma acicola*	Wolf and Barbour (1941)	Sexual	USA	*Pinus* spp.	It was recognized that the pathogen was better suited in the Dothideaceae and therefore the fungus was moved to the genus *Systremma* and all the above names for the sexual state synonymized with *S. acicola.*	
1967	*Dothistroma acicola*	Shishkina and Tsanava (1967)	Asexual	–	–	The name *D. acicola* was incorrectly assigned to both *D. pini* and *L. acicola*. The name was not used in subsequent literature.	Due to similarities between symptoms caused by *Dothistroma* and *Lecanosticta* the asexual states of *D. pini* (presently *D. septosporum*) and *L. acicola* were synonymized and renamed as *D. acicola* and furthermore associated with the sexual state *Systremma acicola.*
1972	*Mycosphaerella dearnessii*	Barr (1972)	Sexual	USA	–	*Systremma acicola* was synonymized with *Mycosphaerella dearnessii*. *Mycosphaerella dearnessii* was assigned as the type for *Mycosphaerella* subgenus *Mycosphaerella* section *Caterva.*	MycoBank accession number: 318138.
1996	*Eruptio acicola*	Barr (1996)	Sexual	–	–	According to Barr (1996), *Mycosphaerella dearnessii* did not fit the description of *Mycosphaerella* and the new genus *Eruptio* M.E. Barr was erected to accommodate *Eruptio acicola* and *Eruptio pini* (sexual state of *Dothistroma septosporum*).	The validity of the genus *Eruptio* was questioned as *Lecanosticta acicola* phylogenetically clusters with other *Mycosphaerella* anamorphs (Crous *et al*. 2001). *Eruptio* is not widely used in literature.
2012	*Lecanosticta acicola*	Quaedvlieg *et al*. (2012)	Both	Europe and North America	*Pinus* spp.	As it was recognized that the genus *Mycosphaerella* should only be used with the anamorphic genus *Ramularia* (Crous *et al*., 2007; Crous, 2009)*, Lecanosticta acicola* was selected as the valid name and type of the genus under the one fungus = one name rule. An epitype was designated for *L. acicola* in this study (Quaedvlieg *et al*., 2012).	MycoBank accession number: 255702 Epitype CBS H‐21113, Ex‐epitype CBS 133791.
Other *Lecanosticta* species							
1984	*Lecanosticta cinerea*	Evans (1984); Marmolejo (2000)	Asexual	Honduras	*Pinus* sp.	The species name was proposed as the correct name for *Gloeocoryneum cinereum*.	This name is not validly published (Marmolejo, 2000) as no basionym was established. The new combination *Leptomelanconium pinicola* was proposed because it had previously been established that *Gloeocoryneum cinereum* is a synonym of *Stilbospora pinicola* and that the genera *Gloeocoryneum* and *Leptomelanconium* have the same characteristics (Marmolejo, 2000).
1984	*Lecanosticta gloeospora*	Evans (1984)	Asexual	Mexico, Iturbide‐Galeana, Nuevo León	*P. pseudostrobus*	The second valid species in the genus to be described based on morphological characteristics.	MycoBank accession number: 106975 Holotype IMI 283812 Ex‐type IMI 283812.
2000	*Lecanosticta longispora*	Marmolejo (2000)	Asexual	Mexico, Nuevo León	*P. culminicola*	The third species described in the genus based on morphological characteristics.	No type was assigned but Quaedvlieg *et al*. (2012) epitypified the species in 2012.
2012	*Lecanosticta longispora*	Quaedvlieg *et al*. (2012)	Asexual	Mexico, Nuevo León	*P. culminicola*	The first phylogenetic study to include this species. An epitype *L. longispora* was designated here.	MycoBank accession number: 466255 Epitype CBS H‐21111, Ex‐epitype CBS 133602.
2012	*Lecanosticta brevispora*	Quaedvlieg *et al*. (2012)	Asexual	Mexico	*Pinus* sp.	This isolate is the fourth species described in the genus based on phylogenetic inference and morphology.	MycoBank accession number: 801940 Holotype CBS H‐21110, Ex‐type CBS 133601.
2012	*Lecanosticta guatemalensis*	Quaedvlieg *et al*. (2012)	Asexual	Guatemala	*P. oocarpa*	The ex‐type of *L. guatemalensis* (IMI281598) was initially described as *L. acicola* based on morphological characteristics (Evans, 1984). The isolate was phylogenetically delineated as a new species in 2012 and subsequently described (Quaedvlieg *et al*., 2012).	MycoBank accession number: 801941 Holotype CBS H‐21108, Ex‐type IMI 281598.
2018	*Lecanosticta jani*	van der Nest *et al*. (2019)	Asexual	Guatemala, Nicaragua	*P. maximinoi, P. oocarpa, P. tecunumanii*	New species described based on phylogenetic and morphological data.	MycoBank accession number: 826875 Holotype PREM 62185, Ex‐type CBS 144456.
2018	*Lecanosticta pharomachri*	van der Nest *et al*. (2019)	Asexual	Guatemala, Honduras	*P. oocarpa, P. tecunumanii*	New species described based on phylogenetic and morphological data.	MycoBank accession number: 826876 Holotype PREM 62188, Ex‐type CBS 144448.
2018	*Lecanosticta tecunumanii*	van der Nest *et al*. (2019)	Asexual	Guatemala	*P. tecunumanii*	New species described based on phylogenetic and morphological data.	MycoBank accession number: 826877 Holotype PREM 62191, Ex‐type CBS 144450.
2018	*Lecanosticta variabilis*	van der Nest *et al*. (2019)	Asexual	Guatemala, Honduras	*P. caribaea, P. maximinoi, P. oocarpa*	New species described based on phylogenetic and morphological data. The ex‐type was initially described as *L. acicola* by Evans (1984) based on morphological observations.	MycoBank accession number: 826878 Holotype PREM 62195, Ex‐type CBS 144453 = IMI 281561.

*Lecanosticta acicola* is the oldest known species in the genus and owes its notoriety to the disease of long leaf pine, which it was first associated with, in the southeastern USA (Chapman, 1926; Hedgcock, 1929). Although the pathogen was identified in Central America based on morphological characteristics (Evans, 1984), it is now recognized as a Northern Hemisphere pathogen for which phylogenetic analyses of the translation elongation factor 1‐α gene (*TEF 1*) sequences have revealed three distinct lineages (van der Nest *et al*., 2019). One of these lineages includes isolates from Canada, the northern parts of the USA (Maine, Michigan, New Hampshire, Vermont and Wisconsin) and Central and Northern Europe (Austria, Croatia, Czech Republic, Estonia, Germany, Italy, Lithuania, Slovenia, Switzerland) (van der Nest *et al*., 2019). A second lineage includes isolates from China, Colombia, France, Japan, Spain, South Korea and the southern part of the USA (Mississippi) (van der Nest *et al*., 2019). A third lineage includes isolates only from Mexico (van der Nest *et al*., 2019).

The eight other species described in *Lecanosticta* during the course of the past 35 years are present only in Mesoamerica (Tables 1 and 2) (Evans, 1984; Marmolejo, 2000; Quaedvlieg *et al*., 2012; van der Nest *et al*., 2019). Evans (1984) recognized considerable morphological variation amongst his collections of *L. acicola.* In that study, he described a second species, *L. gloeospora* from *Pinus pseudostrobus* in Mexico, and the fungus remains known only from Mexico on this host (Evans, 1984; Marmolejo, 2000). The novelty of this species was recently validated using DNA sequence data (van der Nest *et al*., 2019).

*Lecanosticta longispora* was first described based on morphological features from *P. culminicola* in Nuevo León, Mexico (Marmolejo, 2000). This species was characterized in a phylogenetic study by Quaedvlieg *et al*. (2012), and was distinguished from *L. acicola* based on differences in the *TEF 1* and β‐tubulin 2 (*BT 2*) gene sequences. That study was the first to delineate species of *Lecanosticta* based on phylogenetic inference (Quaedvlieg *et al*., 2012). These authors included several samples from Central America that had previously been identified as *L. acicola,* as well as the collection used by Marmolejo (2000) to typify *L. longispora*. In their phylogenetic analyses (Quaedvlieg *et al*., 2012), *L. acicola* was not identified from Central America but two new species, *L. brevispora* and *L. guatemalensis*, were described (Tables 1 and 2)*.*

Evans (1984) observed that ecotypes or morphotypes exist amongst isolates of *L. acicola* in Central America*,* depending on the altitude and hosts from which the isolations were made. He therefore hypothesized that Central America could be the centre of origin of *Lecanosticta*. This was later supported by analysis of *TEF 1* sequence data that revealed high genetic diversity in this geographical region (Janoušek *et al*., 2016). An extensive collection of isolates from Central America was recently studied using a phylogenetic approach (van der Nest *et al*., 2019). Interestingly, *L. acicola* was not identified amongst isolates from Guatemala, Nicaragua or Honduras. Furthermore, the isolates considered to be *L. acicola* by Evans (1984) were sequenced and identified as *L. guatemalensis* and a new species, *L. variabilis* (van der Nest *et al*., 2019, Table 1). *Lecanosticta brevispora* was identified in Guatemala and Honduras on *Pinus oocarpa* and *P. pseudostrobus* (Table 1), expanding the host range and distribution for that species. Likewise, *L. guatemalensis* was also identified in Guatemala, Honduras and Nicaragua on *P. caribaea, P. oocarpa* and *P. tecunumanii* (Table 1)*.* The study of van der Nest *et al*. (2019) introduced four new species, including *Lecanosticta jani* from Guatemala and Nicaragua, *L. pharomachri* from Guatemala and Honduras, *L. tecunumanii* from Guatemala and *L. variabilis* from Mexico, Guatemala and Honduras (van der Nest *et al*., 2019). Although Central America could not be confirmed as a centre of origin of *L. acicola,* the diversity of species recognized by van der Nest *et al*. (2019) suggests strongly that Mesoamerica is a centre of diversity for *Lecanosticta*.

With only one exception, which is probably a taxonomic incongruity, *Lecanosticta* species are all associated with *Pinus* species. Petrak (1954) described *Phragmogloeum gaubae* on *Callistemon sieberi* in Australia (Petrak, 1954). von Arx (1983) attempted to reduce various species with overlapping characteristics to fewer genera and found that *Phragmogloeum* had the same morphological characteristics as *Lecanosticta.* He proposed the new combination *Lecanosticta gaubae*. After the genus *Eruptio* was erected to accommodate *Lecanosticta acicola* and *Dothistroma septosporum* (Barr, 1996), *Lecanosticta gaubae* was transferred to that new genus (Crous, 1999). The genus *Eruptio* was further evaluated and it was found that *L. acicola* and *D. septosporum* were not congeneric (Crous, 2009). Consequently, *Lecanosticta* was selected as the correct name for *Eruptio acicola* following the one fungus one name convention (Crous *et al*., 2009; Hawksworth *et al*., 2011). Because *Eruptio gaubae* is morphologically similar to *Lecanosticta*, phylogenetic analyses are required to resolve this taxonomic confusion.

*Lecanosticta acicola* is the only species in the genus known to be a significant pathogen. This is particularly important because it is spreading rapidly in Europe and the northeastern parts of North America. Therefore, all data collected over time regarding *Lecanosticta* pertain to the organism that was assigned the name *L. acicola*, and the remainder of the review will focus on this species. However, it is relevant to recognize that other species of *Lecanosticta* cause symptoms similar to those of *L. acicola* and that they have the potential to emerge as pine pathogens if they were accidentally moved to new environments. They would then be recognized as members of a complex of BSNB pathogens.

## Symptoms of Brown Spot Needle Blight

**Figure 2:**
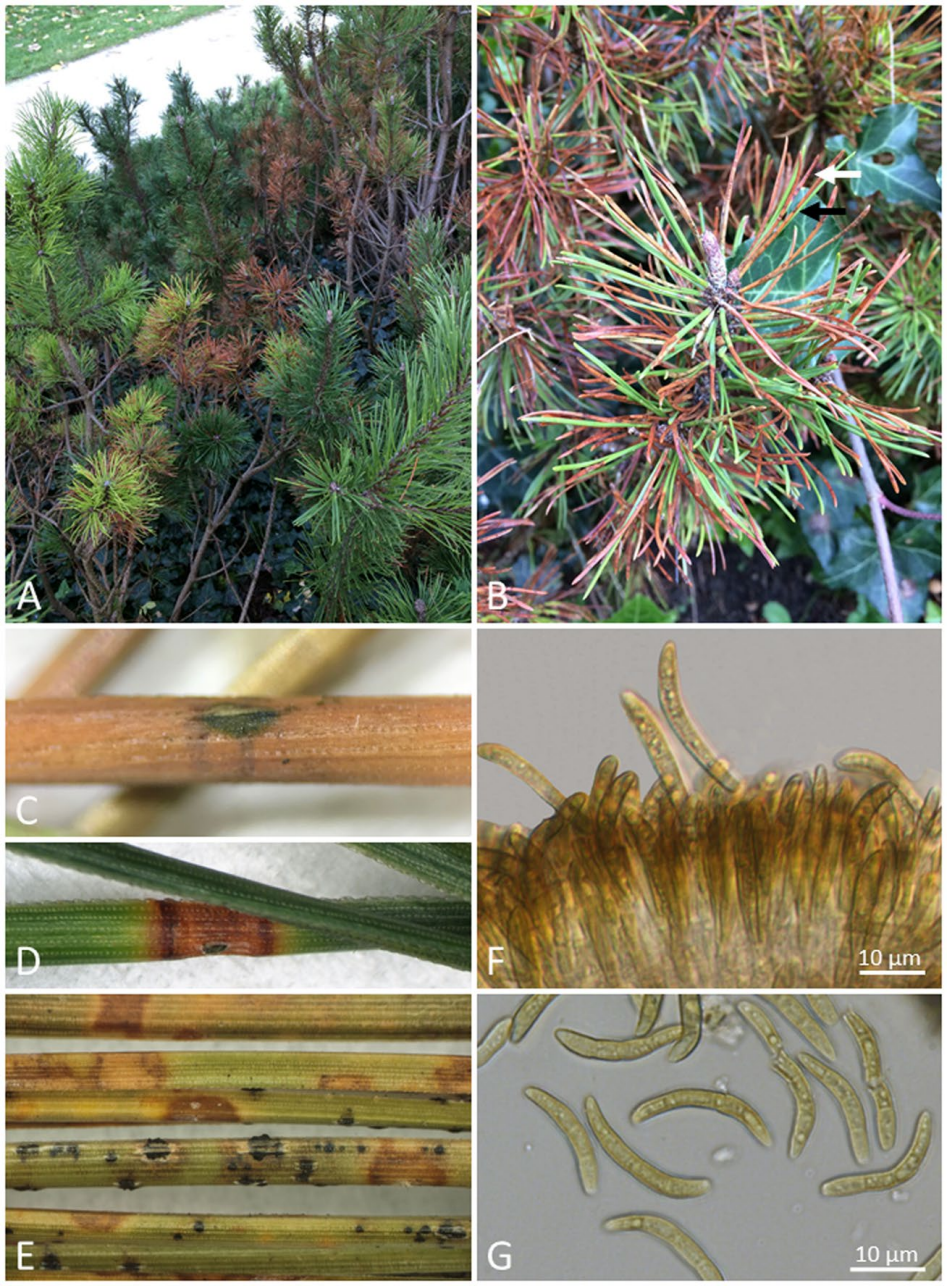
Symptoms of *Lecanosticta acicola.* (A) *Pinus mugo* in Austria displaying symptoms of both brown spot needle blight (BSNB) and Dothistroma needle blight (DNB) on the same branches. (B) Both the characteristic brown spots associated with BSNB (black arrow) and the red banding associated with DNB (white arrow) can be observed. (C)–(E) Symptoms of BSNB vary from only brown spots as observed on *P. mugo* (C) to distinct brown bands as observed on *P. radiata* (D) to irregular mosaic spots as observed on *P. palustris* (E)*.* (F) *Lecanosticta acicola* conidiogenous cells giving rise to conidia on malt extract agar. (G) *Lecanosticta acicola* septate conidia with verruculose surfaces and truncate bases.

Symptoms of infection can vary depending on the host species affected. Typically, a small and yellow, sometimes light grey‐green or reddish brown, irregular circular spot, with defined margins, appears at the point of infection (Hedgcock, 1929) (Fig. 2C–E). These spots soon become brown as the infections mature and they are often surrounded by a yellow halo (Skilling and Nicholls, 1974). In severe cases, infections can occur on several parts of a needle, leading to more rapid necrosis (Fig. 2E). The characteristic brown spots are the first conspicuous symptoms on the pine needles and this has led to the common name ‘brown spot needle blight’ proposed by Siggers (1932). These brown spots can also appear resin‐soaked depending on the host species (Skilling and Nicholls, 1974). In some cases, as has been reported in *P. strobus*, symptoms may only be displayed as chlorosis of the needles without banding (Broders *et al*., 2015). Infected needles die from the apex to the base (Fig. 2B) and they are eventually shed from the trees (Hedgcock, 1929; Skilling and Nicholls, 1974). Usually only the second‐ and third‐year needles are affected, leaving healthy new growth at the tips of the branches. The new growth tips are then infected in the subsequent season by inoculum on older needles (Skilling and Nicholls, 1974). Generally, infection is more severe in the lower parts of the canopy and then progresses upwards in the trees (Sinclair and Lyon, 2005; Skilling and Nicholls, 1974).

An asymptomatic phase in which *L. acicola* establishes within needles can last several days (Setliff and Patton, 1974) to 3 months (Skilling and Nicholls, 1974). This is dependent on the strain of the pathogen (Kais, 1972) and length of the wet season. This delay in symptom development could lead to the accidental movement of infected plants to new areas.

The symptoms of BSNB (Fig. 2) can easily be confused with those of DNB, which is caused by *Dothistroma septosporum* and *D. pini* (Barnes *et al*., 2004, 2016). On some host species, symptoms of DNB are similar to those of BSNB (Fig. 2B) but rather than the characteristic brown discoloration and spots, a distinct red band forms around the point of infection in the case of DNB (Pehl and Cech, 2008). However, in some cases the characteristic red banding pattern associated with DNB is not formed or alternatively the red bands are sufficiently dark to give a false impression of brown spots. This can easily lead to incorrect pathogen diagnoses (Barnes *et al*., 2016; Petrak, 1961).

## Life Cycle

*Lecanosticta acicola* can occur in either its asexual or sexual state (Fig. 1) (Siggers, 1939). The pathogen overwinters in acervuli (asexual) (Fig. 1Aa) or ascostromata (sexual) (Fig. 1Ba) in the dead tissue of either dead or living pine needles. It can also overwinter as vegetative mycelium in the infected needles that remain attached to the host (Siggers, 1944). Conidia are released in gelatinous masses (Fig. 1Ab) or ascospores are released from asci in ascostromata (Fig. 1Bb) on the needles when the light, temperature and humidity are favourable (Kais, 1975; Tainter and Baker, 1996).

Conidia begin to germinate on the needle surfaces by developing one to four germ tubes, depending on the number of cells in the conidia (Setliff and Patton, 1974). It is uncertain whether the germ tubes are attracted to the stomata, or whether they grow randomly over the needle surface (Patton and Spear, 1978; Setliff and Patton, 1974). Light plays an indirect, but essential role in the infection process as it stimulates the opening of stomata, allowing the germ tube to penetrate the needle (Fig. 1c) (Kais, 1975). Infections can also occur through wounds (Kais, 1978). Once a germ tube enters the stomatal antechamber, it increases in diameter and becomes thick‐walled and melanized (Patton and Spear, 1978). Appresoria, such as those found in *Dothistroma* (Gadgil, 1967), have never been seen (Patton and Spear, 1978).

Once the mesophyll tissue has been invaded by *L. acicola* mycelium, conidiomata begin to form. These begin to integrate with the needle tissue and increase in size until they are visible to the naked eye (Wolf and Barbour, 1941). The conidiophores produce conidia towards the leaf exterior (Evans, 1984), which exerts pressure on the needle epidermis. This causes the epidermis to rupture, leaving a flap that partly covers the conidiomata (Wolf and Barbour, 1941). The conidia are released from the conidiomata during wet weather and the disease cycle is repeated.

In the case of the sexual state, asci are formed within the ascostromata on necrotic distal parts of living needles or on dead needles (Henry, 1954; Jewell, 1983). Ascospores are released from asci and dispersed through wind and rain. Asci and ascospores develop more rarely than conidia and have been reported only from Nicaragua, Honduras, Colombia and the southern parts of the USA (Table 1) (Evans, 1984; Henry, 1954; Kais, 1971; Luttrell, 1949; Siggers, 1944). The reports from Nicaragua and Honduras probably represent species other than *L. acicola.*

## Toxin Production

Many plant pathogenic fungi have adapted to produce toxic secondary metabolites in their plant hosts and these could influence colonization and sporulation, as has been seen in *D. septosporum* (Kabir *et al*., 2015). *Lecanosticta acicola* is known to produce the toxic compounds LA‐I and LA‐II, which are heat‐resistant and non‐host specific phytotoxins (Yang *et al*., 2002, 2005). The two compounds interact with the host independently and do not promote or inhibit the interaction of one another  (Yang *et al*., 2002). Different *Pinus* species have different reactions to LA‐I and LA‐II. When rooted cuttings of *P. thunbergii* were exposed to the toxin, they showed little sensitivity to it. In contrast, when *P. elliottii* and *P. taeda*, both highly susceptible to BSNB infection, were exposed to the toxin, the results showed high sensitivity to LA‐I (Ye and Qi, 1999). It seems likely that these toxins are involved in the destruction of mesophyll tissue of the pine needles at the point of infection (Jewell, 1983).

## Biology and Dissemination

Conidia and ascospores are released throughout the year at temperatures ranging from –5.5 to 28 °C (Kais, 1971; Siggers, 1944; Wyka *et al*., 2018). However, warm and wet weather is particularly conducive for the development of BSNB, irrespective of whether infection takes place by sexual or asexual spores. The conidia do not germinate below 5 °C, although most survive this temperature and commence germination once the temperature increases (Siggers, 1944). At the other extreme, tolerance to high temperature was found to vary depending on the strain of *Lecanosticta* involved. It was shown that conidia of isolates from the northern parts of the USA could not germinate at 32 °C, whereas cultures isolated from the southern parts of the USA, as well as China, had a germination success of 80% at the same temperature (Huang *et al*., 1995). This physiological distinction is reflected in population genetic studies which define two lineages of the pathogen in the USA (Janoušek *et al*., 2016). The success of the pathogen may therefore be a result of isolates in each lineage adapting to local temperature conditions.

The maximum temperature for the germination of *L. acicola* conidia is 35 °C (Siggers, 1944). It was also found that high humidity pre‐ and post‐infection is required for high levels of infection (Kais, 1975). The optimal temperature for infection to occur is 30 °C during the day and 21 °C at night, and Kais (1975) showed that these temperatures gave positive results in inoculation trials.

Conidia are dispersed predominantly by rain splash to adjacent trees, and they contribute significantly to rapid disease build‐up in pine stands (Tainter and Baker, 1996). High levels of conidial dispersal were recorded during the rainy season in the USA, especially between late spring and summer, as well as when there were rain spells after a long period of dryness (Kais, 1971). In other reports, conidial production and dispersal were recorded throughout the year (Siggers, 1944). Dispersal was not influenced by the temperature range but conidial release was connected to rainfall patterns. In Wisconsin, two peaks of conidial release were recorded, with the first peak in early summer when young pine needles are present and the second in late summer (Skilling and Nicholls, 1974), which was similar to that found in the northeastern USA (Wyka *et al*. 2018). In Japan, it was found that conidia were produced by the pathogen from early spring to autumn with peak dispersal in mid‐summer. However, for a second year of infection, the dispersal was most abundant from late summer to mid‐autumn the following year (Suto, 2002). A study in Fujian province (China) showed that the greatest number of conidia were detected between early spring and mid‐summer and again in late summer to late autumn in *Pinus elliottii* plantations (Li *et al*., 1987). It consequently appears that conidial dispersal varies depending on the rainfall season in any particular geographical region.

Spore traps in several studies failed to capture ascospores (Kais, 1971; Siggers, 1939; Wyka *et al*., 2018). It was found, however, that conidia could be dispersed to a distance of up to 60 m (Wyka *et al*. 2018). A recent investigation of the dispersal of *Dothistroma*, where the mechanisms of conidial and ascospore dispersal are similar to those in *L. acicola*, showed that conidia could be naturally disseminated over more than 1 km (Mullett *et al*., 2016). The assumed distance of dispersal in *L. acicola* may, consequently, be similar*.*

The ascospores of *L. acicola* are forcibly expelled into the air (Wolf and Barbour, 1941) and dispersed by wind currents (Kais, 1971) or rain splash driven by wind (Siggers, 1939). Ascospores can also be released during periods of fog, rain and dew (Tainter and Baker, 1996). Ascospores were recorded in the USA mainly during periods when temperatures were above 15 °C and are found in late summer to autumn. Small numbers of ascospores were detected when temperatures were below 10 °C (Kais 1971).

The main component that facilitates spread of conidia and ascospores is moisture, but other factors may also aid in their dispersal. Insect dissemination was suggested as a mechanism of conidial spread when two Lepidopteran wing scales were found to have conidia attached to them (Skilling and Nicholls, 1974). Given the biology of *L. acicola*, it seems unlikely that insects are involved in its dissemination. It has also been suggested that animals grazing in forests might aid in dissemination of the conidia when spores stick to their coats or hooves (Skilling and Nicholls, 1974; Tainter and Baker, 1996). Again, this mode of dissemination seems unlikely to be particularly important.

Anthropogenic movement of infected plant material has contributed to the dissemination of many tree pathogens (Wingfield *et al*., 2015). This has been clearly demonstrated for *Dothistroma septosporum* (Barnes *et al*., 2014), which has a biology very similar to that of *L. acicola*. A study that used microsatellite markers has demonstrated that two separate lineages of *L. acicola* have most likely been introduced into Europe from North America (Janoušek *et al*., 2016). Long distance dispersal of *L. acicola* is, therefore, likely to be the result of anthropogenic movement of infected plant material. This would not include seed transmission as *L. acicola* conidia cannot survive on a pine seed's surface longer than 30 to 34 days and it is thus not considered seed‐borne (Jianren and Chuandao, 1988).

## Disease Management

Several measures have been suggested to prevent BSNB during plantation establishment. The most effective is to plant disease‐free seedlings of superior quality (Cordell *et al*., 1990; Skilling and Nicholls, 1974). It is also advisable to avoid establishing new plantations alongside old, infected pines that could potentially serve as reservoirs of inoculum (Tainter and Baker, 1996). For natural pine stands, the application of thinning treatments was investigated as a silvicultural practice against pine needle diseases (McIntire *et al*., 2018). This practice, conducted on native stands of *P. strobus* in the USA, showed promise in reducing the fungal load of *L. acicola*, resulting in reduced severity of the disease over time in stands already infected with the pathogen (McIntire *et al*., 2018). This practice is recommended as a preventative measure in stands that are at risk of infection by *L. acicola* and other pine needle pathogens (McIntire *et al*., 2018).

Pruning of infected pines can contribute to the spread of BSNB if it is conducted during rainy or wet periods. This is because conidia are exuded during these conditions and can attach to the pruning shears, providing a means of spread from infected to healthy trees (Skilling and Nicholls, 1974). Cutting blades should be cleaned during pruning and clipped needles and shoots should be removed (Kais, 1978). In the case of infection on *Pinus palustris*, which begins growth as a grass stage, stimulation of growth during the first 3 years of growth reduces the levels of infection (Tainter and Baker, 1996). Because this treatment is economical, effective and environmentally safe, it is widely used in the southeastern USA (Cordell *et al*., 1990), where BSNB occurs on *P. palustris*.

Breeding for resistance to *L. acicola* has been successfully used to reduce the impact of the disease on *P. palustris* in Alabama. The source population of these trees found in southwestern Alabama was used in breeding programmes (Snyder and Derr, 1972) where seed was made available to the public (Phelps *et al*., 1978). Since 1982, resistant phenotypes of *P. elliottii* have also been selected for in plantations affected by BSNB in the Fujian province in China. Over time, and using artificial inoculations, resistant clones were selected and resistant seed orchards were established (Ye and Wu, 2011).

Fungicide treatment can protect pine seedlings from infection by *L. acicola.* For example, when *P. palustris* was sprayed with fungicide, the seedlings displayed increased diameter growth in a single growing season, compared to untreated plants (Siggers, 1932)*.* Seedlings, seed orchard trees and Christmas tree plantations have been protected by Bordeaux mixture of copper sulphate and lime, which inhibits conidial germination, by a benomyl root treatment or by ferbam (Fermate^®^). Chlorothalonil, a broad‐spectrum organochlorine pesticide (products include Bravo^®^, Daconil^®^ and Maneb^®^), has also been applied to provide efficient control against BSNB. Chlorothalonil is also very effective against *Lophodermium* needle cast, which could be advantageous when both pathogens are present (Cordell *et al*., 1990; Kais *et al*., 1986; Skilling and Nicholls, 1974). Practical details and recommendations concerning fungicide treatment can be found in Skilling and Nicholls (1974). However, the use of chemicals is not considered a desirable solution for disease control due to negative environmental factors and many of these treatments are no longer available.

Controlled burning in pine forests can eliminate competing vegetation and reduce the impact of needle pathogens, especially in *P. palustris* where a grass stage is relevant (Barnett, 1999; Chapman, 1932)*.* This pine species is completely adapted to survive fires as it concentrates all its energy into root development during the first 5 years of growth (Chapman, 1932). Siggers (1934) showed that a single controlled fire can significantly decrease BSNB in *P. palustris* until the next season and that during the initial growth stage, before seedlings begin to increase in height, a winter burn every 3 years is the most beneficial for disease control. The efficacy of controlled burns differs depending on the *Pinus* spp. involved and on the ability to tolerate fire damage.

In countries and regions where *L. acicola* is a quarantine organism, it is suggested that complete eradication of diseased trees or pine stands should be performed once the disease is detected (Pehl and Cech, 2008). This is achieved by felling and burning of infected trees and litter found under infected trees (Sosnowski *et al*., 2009). In Lithuania, for instance, after positive identification of the pathogen in the Curonian Spit in 2009 and 2010, effective eradication measures were implemented (Markovskaja *et al*., 2011). Due to this rapid action, the disease has remained under control in that country and is under constant monitoring by the state plant service of the Ministry of Agriculture of Lithuania (https://gd.eppo.int/taxon/SCIRAC/distribution/LT). Eradication efforts are, however, not always effective, and the best preventative method is to limit the movement of plant material across borders and between regions. As new knowledge is emerging regarding different genetic entities of the pathogen, including strains of different mating types (Sadiković *et al*., 2019), the importance of avoiding new introductions is becoming increasingly obvious.

## Host Range, Host Susceptibility and Geographic Distribution

In an effort to consolidate 140 years of literature with regards to the geographical distribution and host range of *L. acicola*, a detailed list of these data has been compiled (Table 1). This shows that the pathogen has been reported in 31 countries and on 53 pine species and pine hybrids. The majority of the host records on native and non‐native trees are from the Americas, followed by Europe. The pathogen has not been found in Africa, Australia or New Zealand and in South America it is known only in Colombia. Of the 69 reports of the pathogen (Table 1), 31 were made in the last decade (2009–2019). This suggests that incidences of the pathogen are most likely increasing.

In North America, the first report of *L. acicola* was in 1876 on native *Pinus echinata* as *Cryptosporium aciculum* (de Thümen, 1878). Since then, the pathogen has been reported in the USA on several susceptible species, including non‐native *P. caribaea* and *P. pinea*, and native *P. elliottii*, *P. echinata*, *P. glabra*, *P. ponderosa*, *P. rigida*, *P. taeda* and *P. virginiana* (Hedgcock, 1929; Siggers, 1944; Sinclair and Lyon, 2005; Webster, 1930) as well as on regionally planted exotic species such as *P. attenuata*, *P. coulteri*, *P. muricata* and *P. sabiniana* (Siggers, 1944). *Pinus palustris* seedlings are the most severely affected, largely due to the grass stage associated with early growth and where BSNB can cause complete defoliation (Siggers, 1934). Here it can result in mortality reaching 50% and higher in the southeastern USA (Cordell *et al*., 1990). New reports of *L. acicola* causing damage on *P. strobus* have emerged since 2005 in the northeastern USA and Canada and these have been attributed to changes in precipitation and climate in the regions (Broders *et al*., 2015; Wyka *et al*., 2017, 2018). *Lecanosticta acicola* is also recognized as a component of a complex of pathogens that cause white pine needle damage (WPND) in this region (Broders *et al*., 2015). Additionally, the pathogen has been reported on *P. banksiana* and *P. contorta* var. *latifolia* in Canada (Laut *et al*., 1966).

*Lecanosticta acicola* has been reported from 17 European countries (for a complete list of records see Table 1). The pathogen was first recorded in northern Spain in 1942 (Martínez, 1942), where it still occurs on *P. radiata* (Ortíz de Urbina *et al*., 2017). In southwest Europe, *L. acicola* has caused severe defoliation of *P. radiata* × *P.*  *attenuata*, leading to the felling of 100 ha in the 1990s (Lévy, 1996). *Lecanosticta acicola* is spreading through the valleys in the Alps in Switzerland (Holdenrieder and Sieber, 1995), Austria (Cech, 1997; Hintsteiner *et al*., 2012), Italy (La Porta and Capretti, 2000) and Slovenia (Jurc and Jurc, 2010; Sadiković *et al*., 2019), which can be attributed to high humidity in deep valleys or the proximity of lakes. In Europe, *L. acicola* often infects *P. mugo*, a susceptible species on which it has recently caused severe outbreaks in Austria (https://gd.eppo.int/reporting/article-5139). It also infects other pine species such as *P. sylvestris* and *P. nigra.* The pathogen has been recorded in several peat bog sites in southern Bavaria (Germany) and southern Bohemia (Czech Republic). These locations are naturally humid throughout the year and the susceptible pine species *P. mugo* and/or *P. uncinata* subsp*. uliginosa* can be heavily infected, leading to considerable mortality. Similarly, *L. acicola* was recorded in the Baltic states (Drenkhan and Hanso, 2009) and, most recently, also in Sweden (Cleary *et al*., 2019). These records usually come from stands close to the sea or, very frequently, from botanical gardens or urban areas.

Other pine species such as *Pinus* × *rhaetica* and *P. ponderosa* have also been affected by *L. acicola* (Adamson *et al*., 2015, 2018). *Lecanosticta acicola* has been present in Croatia on *P. halepensis* for more than 40 years (Milatović, 1976; Sadiković *et al*., 2019). Interestingly, the pathogen was identified only at a single site in Ireland despite large‐scale screening throughout the British Isles (Mullett *et al*., 2018). From all these records, it is reasonable to conclude that *L. acicola* is spreading in Europe in native and non‐native pine species, in plantations and natural forests, and associated with different climatic conditions.

In Asia, BSNB has been reported in China in plantations of non‐native *P. thunbergii*, *P. elliottii* and *P. taeda* where the trees were severely damaged by the pathogen (Huang *et al*., 1995), and on *P. caribaea*, *P. palustris*, *P. clausa* and *P. echinata* that were reported to be susceptible to infection (Li *et al*., 1986). It was suggested that native pines such as *P. taiwanensis*, *P. fenzeliana* and *P. massoniana* were highly resistant to infection (Huang *et al*., 1995; Li *et al*., 1986). BSNB has been reported on native *P. thunbergii* in Japan (Suto and Ougi, 1998) as well as on native *P. thunbergii* in South Korea but the disease was not severe (Seo *et al*., 2012).

Although some species of *Pinus* seem to not be susceptible to infection by *L. acicola*, the pathogen has the potential to overcome host resistance in a favourable environment and expand its host range, as is suggested for *D. septosporum* and *D. pini* (Drenkhan *et al*., 2016). For example, *L. acicola* is rarely reported on native *P. sylvestris* in Europe. Considering the importance of *P. sylvestris* in Europe, it will be important to monitor the presence of the pathogen on this host. Only single incidences of *L. acicola* have been reported on *P. sylvestris* in Austria (Cech and Krehan, 2008), Slovenia (Jurc and Jurc, 2010) and most recently in Estonia (Adamson *et al*., 2018) and Ireland (Mullett *et al*., 2018). In contrast, *L. acicola* is an important pathogen of *P. sylvestris* grown as part of the Christmas tree industry since the 1960s in the USA (Skilling and Nicholls, 1974). This implies that under favourable conditions this host could be infected by the pathogen. Investigations on the impact of DNB on *P. sylvestris* revealed that there is high intraspecific variability of *P. sylvestris* in Europe and that susceptibility of the host to the pathogen varies between individuals (Perry *et al*., 2016a,b) and this could also influence the potential importance of *L. acicola*. Unusually high humidity associated with climate change could increase pathogen pressure on *P. sylvestris* (Perry *et al*., 2016a) and the single incidences in Europe should carefully be monitored. Caution must also be taken when planting susceptible exotic hosts alongside native forests, as this could influence the vulnerability of native forests (Piotrowska *et al*., 2018).

Of the 69 reports of *L. acicola*, only 22 used DNA sequence comparisons for species verification. This is of concern as there might be an over‐ or underestimation of hosts affected by BSNB globally. In Central America, for example, *L. acicola* was reported based on identifications using morphological characters. Because the pathogen has not yet been confirmed as occurring in this region using DNA sequences (Quaedvlieg *et al*., 2012; van der Nest *et al*., 2019), those reports could be erroneous and may represent different species which could possibly cause new outbreaks if not contained in their native environment.

## Molecular Diagnostics and Future Prospects

### Molecular markers used for species identification

**Figure 3:**
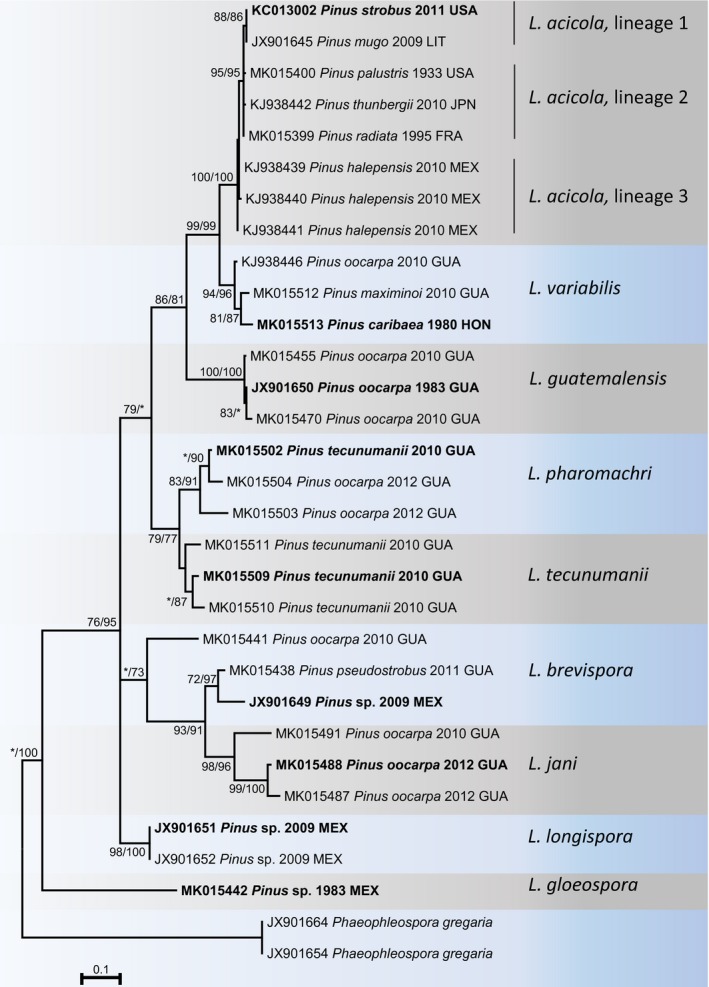
Maximum likelihood (ML) tree representing the nine known species of *Lecanosticta* as well as the three lineages of *L. acicola* generated from the translation elongation 1‐α region. ML bootstrap support (>70%) are indicated first, followed by maximum parsimony (MP) bootstrap support values (ML/MP, * = insignificant value). *Phaeophleospora gregaria* was used as the outgroup taxa. All represented type species are indicated in bold.

Three molecular methods are currently being used to accurately identify *L. acicola*. These include sequencing of various gene regions, an ITS‐RFLP method and a conventional PCR that uses species‐specific primers. The most common of these approaches is comparison of DNA sequences for the ITS gene region (Adamson *et al*., 2015, 2018; Cleary *et al*., 2019; Markovskaja *et al*., 2011; Mullett *et al*., 2018). However, the *TEF 1* (Fig. 3) and *BT 2* gene regions have been recommended to distinguish between species of the Mycosphaerellaceae (Quaedvlieg *et al*., 2012). In order to accurately distinguish between different species of *Lecanosticta,* van der Nest *et al*. (2019) used a multi‐gene phylogenetic approach using sequences for the ITS, *TEF 1, BT 1, MS204* and *RPB 2* gene regions. The outcome was the discovery of four new species, with the ITS and *TEF 1* proving to be the gene regions showing the best amplification success across all species. Pehl *et al*. (2004) developed an ITS‐RFLP method to distinguish between *L. acicola*, *D. septosporum* and ten other plant pathogens. However, whether this method remains valid after the recognition of various new species (van der Nest *et al*., 2019) will need to be established.

Another rapid method allowing for the identification of *L. acicola*, *D. septosporum* and *D. pini* is a conventional PCR that uses species‐specific primers (Ioos *et al*., 2010). These were developed to partially amplify the *TEF 1* gene for *L. acicola* and *D. pini*, and partially amplify the *BT 2* gene region in *D. septosporum* (Ioos *et al*., 2010). Importantly, this method can be used to identify the pathogens directly from infected needles (Adamson *et al*., 2015; Ortíz de Urbina *et al*., 2017; Schneider *et al*., 2019) and is now widely used for preliminary identification of *L. acicola* (Adamson *et al*., 2018; Sadiković *et al*., 2019). A multiplex qPCR was also recently developed to detect *L. acicola* as well as *Dothistroma* species from needles simultaneously using probe‐labelled primers developed by Ioos *et al*. (2010) and Schneider *et al*. (2019), which could become more widely used once that technology is more easily available.

### Population genetic studies

Knowledge regarding the population structure and diversity of pathogens such as *L. acicola* allow for an understanding of migration patterns as well various aspects of their invasion biology. Eleven polymorphic microsatellite markers and mating type primers have been developed for this purpose (Janoušek *et al*., 2014). The first population genetic study using these markers revealed that two lineages of *L. acicola* were introduced into Europe, possibly on two separate occasions (Janoušek *et al*., 2016). These results are similar to an earlier study where RAPD analysis of *L. acicola*, collected in the northern and southern parts of the USA and China, showed that the Chinese population originated from the southern USA and that the collection from the northern USA was unique (Huang *et al*., 1995). A second population genetic study compared populations from Croatia and Slovenia and revealed four distinct populations with possible introductions from other sources within the two countries (Sadiković *et al*., 2019). Currently available knowledge suggests a Northern American centre of origin for this pathogen (Huang *et al*., 1995; Janoušek *et al*., 2016; van der Nest *et al*., 2019) but further sampling and analyses are required to support this hypothesis. In the population genetic study of Janoušek *et al*. (2016), the microsatellite markers amplified poorly for the *L. acicola* isolates from Mexico and Central America. A later study (van der Nest *et al*., 2019) showed that these isolates were *L. variabilis*, a new and recently described species.

The study by Janoušek *et al*. (2014) showed that *L. acicola* is heterothallic and that two individuals, one with a *MAT1‐1‐1* idiomorph and the other with a *MAT1‐2* idiomorph, are needed for sexual reproduction to occur. Consequently, to understand whether sexual recombination might occur in a region, it is important to have a knowledge of the mating type idiomorph distribution. Mating type primers that amplify the *MAT1‐1‐1* and *MAT1‐2* idiomorphs and that tested positive for *Dothistroma* species as well as *L. acicola*, *L. guatemalensis* and *L. gloeospora* have been developed (Janoušek *et al*., 2014). It is, however, not yet known whether these markers will amplify these gene regions for the other, newly described *Lecanosticta* species.

Janoušek *et al*. (2016) considered the global *L. acicola* population and showed that the ratio of mating type idiomorphs in Mississippi, Austria, France and Germany reflected sexual recombination in these regions/countries. In contrast, only asexual reproduction occurs in the Czech Republic and northern parts of America. Using the mating type markers of Janoušek *et al*. (2014), the distribution of MAT1 and MAT2 isolates was detected in studies with isolates from Croatia (Sadiković *et al*., 2019), Estonia (Adamson *et al*., 2015, 2018), Ireland, Portugal, Russia (Mullett *et al*., 2018) as well as Spain (Ortíz de Urbina *et al*., 2017). In Spain, both mating types were detected whereas only single mating types were detected in all other areas studied. However, in Estonia it was suggested that a second introduction of the pathogen occurred since only MAT1 was initially present but that later both mating types were detected in the same region (Adamson *et al*., 2015). In populations with equal ratios of mating types or with both mating types present, sexual reproduction could occur, possibly giving rise to more virulent strains. This emphasizes a need to exercise caution and thus to prevent introduction of new strains into regions where the pathogen is already present.

### Future prospects in the age of genomics

Canada's Michael Smith Genome Sciences Centre has recently released a full genome for a *L. acicola* isolate from France (https://www.ncbi.nlm.nih.gov/assembly/GCA_000504345.2#/def). This genome has not yet been annotated but provides a valuable resource for future studies. Many other genomes of Dothidiomycetes, which have been sequenced and annotated, are available for comparative purposes (de Wit *et al*., 2012; Ohm *et al*., 2012). Annotation of putative genes of the *L. acicola* genome, utilizing knowledge of these other genomes, will provide insights into questions regarding many aspects of the biology of *L. acicola*. Opportunities also now arise to sequence the genomes of other *Lecanosticta* spp. and to compare these in order to better understand their relative importance. It will also be possible to follow the *Dothistroma* example where a transcriptomic study considered which genes are expressed during various stages in the infection of *P. radiata* (Bradshaw *et al*., 2016) and genome sequencing of global representatives of *D. septosporum* revealed that gene copy numbers could play a role in dothistromin production by the pathogen (Bradshaw *et al*., 2019).

## Conclusions

*Lecanosticta acicola* has been known in the southern USA for many decades. Consequently, its life cycle, mode of infection, host susceptibility and strategies to prevent infection, particularly on *P. palustris*, have been extensively studied in that region. Yet there is evidence to show that the pathogen, which now has an extensive host range, is spreading rapidly northwards. The reasons for this host range and geographical expansion require further study. Contemporary knowledge has also shown that there have been two introductions of *L. acicola* into Europe. Consequently, BSNB is becoming a disease of great concern in Europe, where it is increasingly being discovered on both non‐native and native *Pinus* spp. There are many relevant hypotheses to explain the growing importance of BSNB and these include the effects of climate change, emergence of more aggressive strains of the pathogen and anthropogenic processes leading to new introductions. There is clearly a need for increased attention to and studies of *L. acicola*, particularly in Europe.

Recent studies have shown that there are eight species of *Lecanosticta* in addition to *L. acicola.* All of these other species appear to have a Mesoamerican origin. Much of the literature pertaining to *L. acicola* needs to be reconsidered given the fact that a single name has been widely used to refer to what we now know represents numerous cryptic species. *Lecanosticta acicola* identified based on DNA sequence comparisons has not been found in Central America, suggesting a North American centre of origin. Of the 69 reports of *L. acicola*, only 25 from 12 countries have been confirmed using DNA sequence‐based tools (Table 1). Many reports of the pathogen could thus be erroneous and there is an urgent need to resolve this important question.

All the available knowledge regarding BSNB relates to studies on *L. acicola* and these are predominantly from the USA. Nothing is known regarding the relative importance of the remaining eight species of *Lecanostica*. At least some of these are most likely also important pathogens and their relative threat to global forests and forestry needs to be assessed. A concerted effort must be made to prevent their accidental introduction into new regions of the world and as part of this process DNA sequence‐based techniques need to be routinely applied to allow for meaningful identification.

The development of new tools to study *Lecanosticta* spp. and BSNB provides many exciting opportunities to enhance our knowledge of this important group of pathogens. The population structure and diversity of *L. acicola* can now be easily studied in the USA as well as where new invasions occur in Europe, and at levels that were previously not possible. For example, application of the available microsatellite markers will enable a more comprehensive understanding of the pathogen as well as determination of its centre of origin.

Genome sequencing is rapidly becoming cheaper and more readily available, and an isolate of *L. acicola* is already available in the public domain for study. We envisage that all the species of *Lecanosticta* will be sequenced in the relatively near future and many isolates of some species will likely also be studied at this level. These studies, and others relating to the ‘omics’ level, will surely have a substantial impact on our understanding of a group of pathogens that is growing in importance and relevance. Overall, BSNB (including all species of *Lecanosticta*) has the potential to become a pine needle disease of global importance if proper preventative measures for the spread of the causal pathogens are not implemented.

## Accession Numbers

The aligned dataset used to draw Fig. 3 is deposited in TreeBASE (No. S24301).
